# Predictive modelling using neuroimaging data in the presence of confounds

**DOI:** 10.1016/j.neuroimage.2017.01.066

**Published:** 2017-04-15

**Authors:** Anil Rao, Joao M. Monteiro, Janaina Mourao-Miranda, Alzheimer's Disease Initiative

**Affiliations:** a Department of Computer Science, University College London, United Kingdom; b Max Planck University College London Centre for Computational Psychiatry and Ageing Research, University College London, London, United Kingdom

## Abstract

When training predictive models from neuroimaging data, we typically have available non-imaging variables such as age and gender that affect the imaging data but which we may be uninterested in from a clinical perspective. Such variables are commonly referred to as ‘confounds’. In this work, we firstly give a working definition for confound in the context of training predictive models from samples of neuroimaging data. We define a confound as a variable which affects the imaging data and has an association with the target variable in the sample that differs from that in the population-of-interest, i.e., the population over which we intend to apply the estimated predictive model. The focus of this paper is the scenario in which the confound and target variable are independent in the population-of-interest, but the training sample is biased due to a sample association between the target and confound. We then discuss standard approaches for dealing with confounds in predictive modelling such as image adjustment and including the confound as a predictor, before deriving and motivating an Instance Weighting scheme that attempts to account for confounds by focusing model training so that it is optimal for the population-of-interest. We evaluate the standard approaches and Instance Weighting in two regression problems with neuroimaging data in which we train models in the presence of confounding, and predict samples that are representative of the population-of-interest. For comparison, these models are also evaluated when there is no confounding present. In the first experiment we predict the MMSE score using structural MRI from the ADNI database with gender as the confound, while in the second we predict age using structural MRI from the IXI database with acquisition site as the confound. Considered over both datasets we find that none of the methods for dealing with confounding gives more accurate predictions than a baseline model which ignores confounding, although including the confound as a predictor gives models that are less accurate than the baseline model. We do find, however, that different methods appear to focus their predictions on specific subsets of the population-of-interest, and that predictive accuracy is greater when there is no confounding present. We conclude with a discussion comparing the advantages and disadvantages of each approach, and the implications of our evaluation for building predictive models that can be used in clinical practice.

## Highlights

• Definition of confound given from the point of view of predictive modelling.
• Instance Weighting derived for dealing with confounding with continuous targets.
• None of the evaluated methods performs better than a model that ignores confounding.
• Different methods favourably predicted different strata of population-of-interest.
• Predictive accuracy over population-of-interest reduced in presence of confounding.

## 1 Introduction

There has been substantial interest in recent years in using multivariate regression models to predict clinical and psychometric scales from neuroimaging MRI (Stonnington et al., 2010). There remains however, some uncertainty as to how to incorporate variables such as age and gender in predictive modelling (Brown et al., 2012). Such variables, which are highly correlated with the imaging data but which are uninteresting from a clinical perspective, are commonly referred to as ‘confounds’.

In the context of predictive modelling in neuroimaging, there does not appear to be a precise definition of ‘confound’. Even so, the standard approach to dealing with variables such as age and gender, is to ‘regress out’ their contribution to the image data (Dukart et al., 2011, Abdulkadir et al., 2014) before estimating the predictive model. Here, we fit a linear model for each image feature using the confounds as predictors, and consider the residuals to be the image data after ‘adjusting’ for the confounds. The adjusted image data is then used as the input features in the predictive model. The aim is to remove variability in the image features associated with the confounds, thereby improving predictions while also producing a model that can be interpreted as being driven solely by the image data. As an alternative to using adjusted images, we can utilise the confounds by including them as predictors along with the original image features during predictive modelling (Rao et al., 2015). The principle underlying this approach is that all variables should be included in the model and their contribution to the final predictive model will be recovered during model training, without the need for any prior procedure such as adjustment. The resulting model will then explicitly be a multivariate function of the image data and the confounds. Finally, we can choose to perform a ‘matching’ of subjects based on the values of the confounds, rather than alter the modelling procedure using image adjustment or including the confounds as predictors. This is typically employed during binary classification tasks where it is relatively simple to select a subset of subjects that has the same distribution of age/gender in the two groups. Matching, however becomes much more difficult as the number of confounds increases or when the target variable is continuous as in regression tasks. In addition, matching necessarily involves discarding subjects in order to create the matched sample, which can be considered wasteful from a machine learning perspective, whilst also undesirable given the financial and labour costs of acquiring data.

In other disciplines such as epidemiology, the concept of confounding is well established but there the goal is to estimate group differences in the presence of confounding rather than develop predictive models. In this paper, we will use the following explicit working definition for confound, which is motivated specifically by issues that arise with predictive modelling:

**Definition.** For a given data sample *D*, a confound is a variable that affects the image data and whose sample association with the target variable is not representative of the population-of-interest. The sample *D* is then said to be biased (by the confound), with respect to the population-of-interest.

**Fig. 1:**
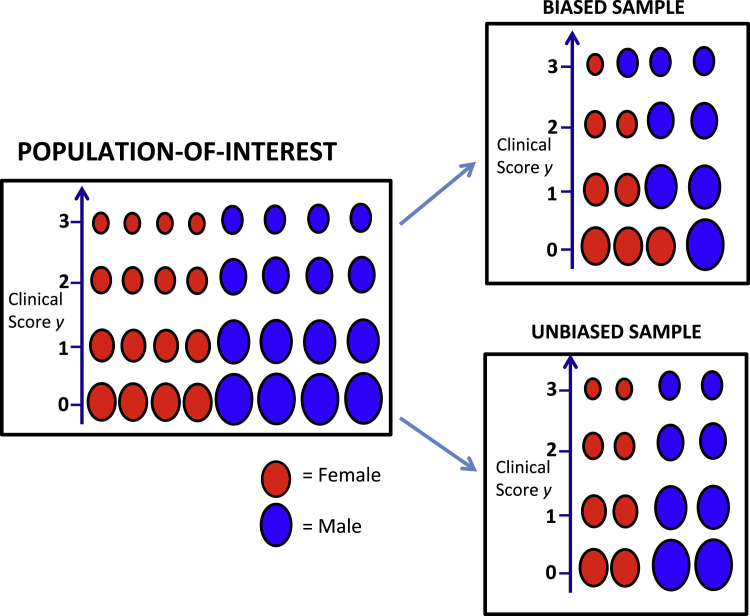
A schematic of an example illustrating biased and unbiased samples from a population-of-interest. Here, the target variable y is a clinical score, and each ellipse represents the brain of a subject, with larger ellipses indicating a larger brain volume. Gender, indicated by red/blue, plays the role of the confounding variable, with males tending to have larger brains than females due to increased head size. In the population-of-interest, each clinical score *y* is equally likely, and overall there is an even distribution of gender. There is also no association between clinical score and gender, as gender is evenly distributed for every clinical score. In the population-of-interest, decreases in brain size are associated with increases in y, and we wish to recover this predictive model of y using samples taking from this population. The biased sample, however, contains a correlation between gender and *y* that is not present in the population-of-interest, with males tending to have higher values of y than the females. In contrast, the unbiased sample has an even split of males and females for each value of the target y, and thus is representative of the population-of-interest.

An important component of the above definition is the idea of a ‘population-of-interest’, which is the population over which we wish to apply the model that is estimated from the data sample *D*. Note that if a variable affects the image data but its association with the target variable *is* representative of the population-of-interest, we would then consider the sample to be *unbiased*, and the variable is not a true confound. While our definition of confound is general, in this paper we will focus on a particular type of confounding where the confound and target variable are independent in the population-of-interest, but the training sample is biased due to a sample association between the target and confound. Such a situation may occur if e.g., we would like our predictive model to predict a clinical score equally well for both male and female subjects across the values of the clinical score, but our training sample shows a significant difference between the values of the clinical score for each gender. In that case, the training sample can be considered as biased by gender, with respect to the population-of-interest. This is illustrated in Fig. 1, where we also show an example of an unbiased sample in this scenario. Note that for unbiased samples, we no longer consider gender to be a confound (by definition) even though it explains variability in the image data.

Our definition differs from the common usage of ‘confound’ with respect to predictive neuroimaging models, where ‘confound’ is often used to describe an uninteresting variable that affects the imaging data, without considering its relationship with the target variable we want to predict. For example, Kostro et al. (2014) use image adjustment to improve the classification accuracy of Huntington's disease when using data acquired from different scanners of subjects with differing age, sex, and total intracranial volume. In that work, the scanner and demographic variables were described as ‘covariates’, although the method used to remove variability in the images associated with the covariates was described as ‘removing confounding effects’ without regard to possible associations between the covariates and the target variable, i.e., a diagnosis of Huntingdon's Disease. A recent work (Wachinger and Reuter,), does not explicitly refer to confounds, but discusses an Alzheimer's Disease classification scenario in which the image data available for training is not all from the same study as that of the test set. Although the differences in image data between the training and test data were characterized using variables such as age and gender, the authors neither explicitly refer to confounding relationships between these variables and the target variable, nor propose a solution that can deal with biased datasets that contain confounds. In Linn et al. (2015), confounding is defined from the perspective of causality and relationships between the potential confound and the target variable are considered. The authors describe and evaluate an algorithm that deals with confounding in classification problems, by essentially weighting observations in a biased training sample to artificially create an unbiased training sample that is representative of the population-of-interest. Although their derivation of the weighting scheme explicitly refers to binary targets, and hence classification problems, it should be noted that similar weighting schemes have been derived outside neuroimaging that are appropriate for the estimation of causal effects with continuous targets (Hirano et al., 2004). However, the authors of Linn et al. (2015) do not consider the prediction of continuous targets such as clinical scores, nor do they investigate the qualitative and quantitative impact of confounding on predictive accuracy.

The overall aim of this paper will therefore be to discuss and evaluate methods for building predictive neuroimaging models using biased training samples that can perform optimally on an unbiased dataset that is representative of the population-of-interest. For a given dataset, this will require us to create biased samples for training, which are then used to predict unbiased samples over which we determine evaluation metrics of predictive performance. Our evaluation framework contrasts with previous works that mention confounding such as Dukart et al. (2011), Kostro et al. (2014), in which either the training samples are unbiased, or the test samples are themselves biased with respect to the population-of-interest. Our framework also facilitates an analysis of how the relationship between confound and target variable affects the distribution of prediction errors in the unbiased test samples. In addition to evaluating image adjustment and the inclusion of confounds as predictors, we will motivate and evaluate the use of ‘Instance Weighting’ that attempts to directly model the relationship between the confound and the target in the biased sample, and uses this to weight training examples in the predictive modelling to improve predictive performance on unbiased data. This approach to dealing with confounding is similar to the algorithm described in Linn et al. (2015), but in contrast to that work, our evaluation focuses on the prediction of continuous targets where the number of features is greater than the size of the training sample.

Section 2 now describes standard approaches for dealing with confounds, while Section 3 motivates the use of Instance Weighting for dealing with confounding through Empirical Risk Minimization. Experiments with image data from the ADNI and IXI databases are presented in Section 4, and we conclude with a discussion of the evaluated approaches, and the implications for building predictive models that are useful in clinical practice.

## 2 Standard approaches for dealing with confounds

We have a sample of *n* observations consisting of image features $g_{i}\equiv (g_{i1},\ldots ,g_{{\mathrm{id}}_{G}})\in R^{d_{G}}$, confounds $c_{i}\in R^{d_{C}}$ and target variables *y*_*i*_ that we wish to predict. These are collected into the corresponding matrices $G\in R^{n\times d_{G}}$, $C\in R^{n\times d_{C}}$ and $y\in R^{n}$. The complete sample 〈G,C,y〉 will be referred to by the symbol D, and the population-of-interest will be denoted by T. We will assume that the confounds c are independent of the target variables *y* in T.

### 2.1 Use images only

If we choose to ignore the confounds C, then we learn the predictive function f

(1) f(g)→y

from the sample 〈G,y〉, using our algorithm of choice e.g., kernel ridge regression. This is the default method if we do not try to control for confounding.

### 2.2 Image adjustment by confounds

Image adjustment aims to remove variability in each image feature associated with the confounds, giving adjusted data that can be considered as having been produced by subjects with identical confound values. For example, if the confound is gender, image adjustment aims to change the image data so that it is as if all subjects were male. If successful, the resulting sample will now be unbiased, and the relationship between the adjusted data and targets can then be learned in the usual manner.

Adjusted images are usually produced by firstly fitting a linear model to each image feature in turn:

(2) gij=c^iβj+ϵij

where $j\in \{1,\ldots ,d_{G}\}$ is the index of the image feature, and c^i is $c_{i}$ augmented with a 1 to account for the intercept term. The adjusted image feature $g_{\mathrm{ij}}^{A}$ for subject *i* at image feature *j* is then given by

(3) gijA=gij−c^iβj.

We can use the following matrix procedure to simultaneously perform a least squares fit of the parameters $\beta_{j}$ for each image feature in Eq. (2), and adjust the features according to Eq. (3):

(4) GA=G−C^(C^TC^)−1C^TG,

where C^ is C augmented with a column of ones. In Eq. (4), the *j*th column of (C^TC^)−1C^TG gives the least squares estimates for $\beta_{j}$, and the above corresponds exactly to the kernel residual forming framework given in Chu et al. (2011). The *i*th row of $G^{A}$ gives the adjusted image data for subject *i*, and we now learn the predictive function

(5) $$f(g^{A})\to y$$

from $\langle G^{A},y\rangle$ using our algorithm of choice.

Note that the model in Eq. (2) is sometimes fit using a selected subset of subjects S, followed by an adjustment of the complete set of data via Eq. (3). This can be performed using the following modification to Eq. (4)

(6) GA=G−C^(C^STC^S)−1C^STGS

where GS,C^S are the rows of G,C^ corresponding to the subjects S. For example, if we are evaluating the performance of a regression/classification model in a training-test paradigm, we may choose to determine the adjustment model using only the training sample, in which case S is the set of training subjects. Adjustment using a subset of the data is also often used in the classification of Alzheimer's disease from grey matter volume images derived from structural MRI, where S is taken to be the set of healthy controls. The motivation for this is that a confound such as gender may affect grey matter volume differently in a subject with Alzheimer's disease to that of a healthy subject. If we were to include diseased subjects in the fitting, this could therefore potentially worsen the adjustment model for the healthy subjects. In this paper, we restrict ourselves to the case of adjusting with all available data as in Eq. (4), as this is the more general case and does not require us to make assumptions about how the effects of a confound on image data change as the value of the target varies.

### 2.3 Incorporating confounds as predictors

In this approach, the confounds are explicitly included as predictors in the model and we learn the predictive function

(7) f(g,c)→y

using the complete data D. Here the confounds are treated in a similar fashion to the image features and allowed to model the target variable in an unrestricted manner. The advantage of this approach is that, in practice, we may not know whether or not the population-of-interest contains associations between c and *y*. For example, it could be that females are more likely to have a higher clinical score than males in our sample, and that this is also true in our population-of-interest, i.e., the sample is unbiased. It may then be advantageous to include c, i.e., gender as a predictor. Alternatively, if the population-of-interest does not contain this association, ie., the sample is biased, including c as predictors should not reduce predictive performance provided the conditional relationship in the sample between the target *y* and the *complete* set of input features {g,c} is representative of the population-of-interest. This approach therefore puts everything into the model and trusts the model training procedure to recover a model that predicts well regardless of possible bias in the training sample.

This approach may run into problems, however, due to the phenomenon known as *covariate shift*, which occurs when the distribution of the predictor features in the training sample does not match the distribution in the population-of-interest. In the presence of confounding, covariate shift will arise because associations between confounds and the target variable in the training sample cause the image data to be unrepresentative of the population-of-interest. If the predictive model is also misspecified, i.e., if the ‘true’ predictive model is not one of the candidate models considered during model fitting, covariate shift will cause the recovered model to focus on particular examples in the training sample rather than the population-of-interest (Shimodaira, 2000). Although a number of different approaches have been proposed in the machine learning literature for dealing with covariate shift e.g., Bickel et al. (2009), Sugiyama et al. (2008), Gretton et al. (2009), Pan et al. (2011), the focus was not on the specific type of covariate shift that occurs due to confounding. Moreover, those methods were generally applied to datasets that did not have the extremely high ratio of feature dimension to training sample size that we typically face in neuroimaging. In A.1 we demonstrate the consequences of covariate shift with a synthetic example in which a single image feature is used to predict a continuous target in the presence of a single confound. There, we show that if the model is correctly specified, including the confound as a predictor gives models that predict unbiased samples equally well, regardless of whether the training sample is biased or unbiased: Even though the biased sample contains a correlation between the confound and the target that is not present in the population-of-interest, including the confound as a predictor does not degrade predictive performance. However, when we repeat the experiment under model misspecification, predictive accuracy is much worse with the biased training sample than with the unbiased training sample. This has practical consequences for performing predictive modelling in neuroimaging in the presence of confounding, where the modelling is typically exploratory, and we do not know the best model ‘a-priori’. Hence, there will always be a degree of model misspecification, and so including confounds as predictors in an attempt to control for confounding may give models that perform poorly on unbiased data. Nevertheless, this approach is often used as a comparator method to other approaches (Kostro et al., 2014) so we consider it in this work.

Although the example in A.1 focuses on models in which the confounds are included as predictors, model misspecification means that, in practice, confounding may degrade the predictive performance of *any* predictive modelling procedure, including the ‘Images Only’ and ‘Adjusted Images’ approaches. This is because even if we do not explicitly include the confounds in the model, we still wouldn't know a priori the appropriate form for the image-features part of the model and so there will be a degree of model misspecification. The combination of model misspecification and covariate shift associated with the confounding will then once again reduce predictive accuracy. In the following section we derive an approach based on ‘Instance Weighting’, which attempts to deal with the specific type of covariate shift associated with confounding in order to reduce the impact of model misspecification. In principle, this approach can then be used with any supervised learning problem where there is confounding in an attempt to improve predictive accuracy.

## 3 Adjusting for confounds using instance weighting

### 3.1 Empirical risk minimization

We firstly describe Empirical Risk Minimization (ERM) which is the standard frequentist framework for supervised learning (Vapnik, 1992). In ERM, we aim to obtain the optimal model $f^{*}$ within a model class F, for the probability distribution $P_{X,Y}$ under a loss function *l* by minimizing the expected risk (Vapnik, 1992):

(8) $$f^{*}=\underset{f\in F}{\arg}\min\int_{(x,y)\in X\times Y}l(f(x),y)\;{\mathrm{dP}}_{X,Y},$$

where x consists of any feature set rather than specifically the image features as described in previous sections. This can be rewritten in terms of the corresponding density P(x,y) as Scholkopf and Smola (2002)

(9) $$f^{*}=\underset{f\in F}{\arg}\min\int_{(x,y)\in X\times Y}l(f(x),y)P(x,y)\;dx\;\mathrm{dy}.$$

We now return to our situation in which we wish to predict targets *y* using image features g and (potential) confounds c, so now x≡(g,c). The optimal function is then given by

(10) $$f^{*}=\underset{f\in F}{\arg}\min\int_{(g,c,y)\in X\times Y}l(f(g,c),y)P^{T}(g,c,y)\;dg\;dc\;\mathrm{dy}$$

where $P^{T}$ refers to the joint density of the image features, confounds, and targets in the population-of-interest. In practice, we do not know the density $P^{T}$, but instead have a training sample of *n* observations. In standard ERM we compute the average loss with respect to the empirical cumulative distribution function of (g,c,y):

(11) $$f^{*}=\underset{f\in F}{\arg}\min\overset{n}{\underset{i=1}{\sum }}\frac{1}{n}l(f(g_{i},c_{i}),y_{i}).$$

The above equation is essentially what was used to fit the least-squares models in A.1, and it ignores any potential bias in the training sample.

### 3.2 Instance weighting

When confounding is present, not only do we not know the full density of the population-of-interest $P^{T}$, but we aim to learn the predictive function using a biased sample from T. In contrast to standard ERM learning, we address bias in the sample by considering it to be a random sample drawn from a different density $P^{S}$ to that of the population-of-interest. We then express $P^{T}(g,c,y)$ in terms of $P^{S}(g,c,y)$ as follows:

(12) $$P^{T}(g,c,y)=\frac{P^{T}(g,c,y)}{P^{S}(g,c,y)}P^{S}(g,c,y)\;=\frac{P^{T}(g|c,y)P^{T}(c,y)}{P^{S}(g|c,y)P^{S}(c,y)}P^{S}(g,c,y)$$

We then make the following important assumption:

(13) $$P^{T}(g|c,y)=P^{S}(g|c,y)$$

which means that given a particular value of the target and the confound variables, the probability density of the image data is the same in $P^{S}$ and the population-of-interest. Effectively we are saying that there is no systematic difference between the image data of a subject drawn at random from $P^{S}$, i.e., the image data in our sample, and the population-of-interest when restricted to subjects with the same combination of target and confound values: It is the difference in the relationship between the targets and the confounds in the two densities that needs to be accounted for. Given this assumption we can write Eq. (10) as

(14) $$f^{*}=\underset{f\in F}{\arg}\min\int_{(g,c,y)\in X\times Y}l(f(g,c),y)\frac{P^{T}(c,y)}{P^{S}(c,y)}P^{S}(g,c,y)\;dg\;dc\;\mathrm{dy}.$$

As with standard Empirical Risk Minimisation, we use estimates for the probability densities giving

(15) f*=argf∈Fmin∑i=1n1n[P^T(ci,yi)P^S(ci,yi)]l(f(gi,ci),yi)

where P^T(ci,yi),P^S(ci,yi) are estimates of the densities $P^{T}(c,y),P^{S}(c,y)$, evaluated at the *i*th training point. We can see that the above expression is similar to that for standard ERM learning in Eq. (11), but now the minimisation is of a weighted version of the loss function over the training set, where the weight associated with training point *i* is equal to [P^T(ci,yi)P^S(ci,yi)]. The weighting therefore scales the contribution of the training point to reflects its density in the population-of-interest T, although now we need to calculate the weights. This weighting of the loss function is similar in spirit to those derived in the machine learning covariate-shift literature e.g., Shimodaira (2000), Sugiyama et al. (2007). A direct application of those methods, however, results in weightings of the form [P^T(gi,ci)P^S(gi,ci)], i.e., they would require an estimate of the ratio between two densities of extremely high dimension due to the inclusion of the image features g. Instead, by use of the factorisation in Eq. (12) and the specific assumption of Eq. (13), we derive the appropriate weights in terms of the joint densities of the target variable and confounds, which are much easier to estimate due to the relatively small number of confounds. Note that as in the weighting approaches of Shimodaira (2000), Sugiyama et al. (2007), we assume that the support of the numerator density is contained in the support of the denominator density in Eq. (15).

As mentioned in Section 1, the focus of this work is the case in which the confounds and targets are independent in the population-of-interest. Under this assumption we can further simplify the weights giving

(16) f*=argf∈Fmin∑i=1n1n[P^T(ci)P^T(yi)P^S(yi|ci)P^S(ci)]l(f(gi,ci),yi)

where we have also factorized the denominator. If we also assume that the marginal distributions of the confounds and targets are identical in T and S, we have

(17) f*=argf∈Fmin∑i=1n1n[P^S(yi)P^S(yi|ci)]l(f(gi,ci),yi).

Finally, note that the form of the predictive function f, i.e., the model, is flexible. For example, we could choose to employ a model in which the predictive function does not depend on c so that the optimal function is given by

(18) f*=argf∈Fmin∑i=1n1n[P^S(yi)P^S(yi|ci)]l(f(gi),yi).

Although this removes the role of the confound from the predictive model, the motivation and derivation of the weighting scheme is still applicable: The weighting will continue to focus the predictions on the population-of-interest T.

### 3.3 Applying instance weighting

In practice, using Instance Weighting proceeds in two stages. In the first stage, the weights

(19) wi=P^S(yi)P^S(yi|ci)

in Eq. (17) need to be determined from the available training data 〈G,C,y〉, which is considered to be a random sample from $P^{S}$. This involves estimating the ratio of the marginal and conditional distributions $P^{S}(y),P^{S}(y|c)$, from the data, and then evaluating Eq. (19) at each training point *i*. In the second stage, we solve the weighted problem given in Eq. (17) or (18), with a choice of loss function *l* that is appropriate for our problem domain. A.2 demonstrates how Instance Weighting improves the predictive accuracy when training with biased samples in our simulated example. It is worth noting that the weights given in Eq. (19) correspond to those given in the causal inference literature for estimating continuous treatment effects (Imai and Ratkovic, 2014).

We now go on to describe our evaluation of all the presented methods for dealing with confounds with real imaging data.

## 4 Experiments with imaging data

### 4.1 Materials

The first dataset consisted of the MP-RAGE images of 592 unique subjects from the Alzheimer's Disease Neuroimaging Initiative (ADNI) database (adni.loni.usc.edu). The ADNI was launched in 2003 as a public-private partnership, led by Principal Investigator Michael W. Weiner, MD. The primary goal of ADNI has been to test whether serial magnetic resonance imaging (MRI), positron emission tomography (PET), other biological markers, and clinical and neuropsychological assessment can be combined to measure the progression of mild cognitive impairment (MCI) and early Alzheimer's disease (AD). Up-to-date information is available at http://www.adni-info.org. The data was preprocessed using SPM12 (http://www.fil.ion.ucl.ac.uk/spm/software/spm12/) and consisted of grey matter segmentation and group-wise registration using Dartel to a study-specific template. The aligned images were transformed to the 2 mm MNI template and smoothed with a Gaussian kernel of 2 mm FWHM. A mask was applied to select voxels that had a probability of being grey matter above 0.025, giving a set of images that provide the 157026 image features g in the matrix G. In our experiments, the image features will be used to predict the MMSE (Mini-Mental State Examination) score which is a measure commonly used to diagnose and assess dementia. The MMSE score is therefore the target variable *y*, and gender will play the role of the confounding variable *c*.

The second dataset consisted of the T-1 images of 580 healthy subjects from the IXI database http://brain-development.org/ixi-dataset/. These images were acquired from 3 different sites with varying scanner properties: Guy's Hospital (Philips 1.5 T), Hammersmith Hospital (Philips 3 T), and the Institute of Psychiatry (GE 1.5 T). The images were preprocessed using SPM8 (http://www.fil.ion.ucl.ac.uk/spm/software/spm8/) and consisted of grey matter segmentation, normalisation to the 2 mm MNI template and smoothing with a Gaussian kernel of 10 mm FWHM. A mask was applied to select voxels that had a probability of being grey matter above 0.05, giving a set of images that provide the 210539 image features g in the matrix G. For the IXI data, the image features will be used to predict the age of the subjects which is therefore the target variable *y*, and acquisition site will play the role of the confounding variable c. Acquisition site has been shown to affect imaging data even when the same make and specification of scanner is used in each site (Focke et al., 2011, Takao et al., 2013), and although is unlikely that we would want to predict age from T1 images in a clinical setting, this dataset still enables us to compare the different approaches for dealing with confounding using real high-dimensional imaging data. Since the IXI data contains a mixture of young and old participants, we restrict the subjects to be those above the age of 47. We also only include subjects from Guy's Hospital and the Hammersmith Hospital as these have the greatest numbers of subjects, so that *c* has two possible values. The resulting initial pool of IXI data consists of 274 subjects.

While we consider each dataset to be confounded by a single discrete variable in our experiments, in practice we often have to deal with datasets with multiple confounds consisting of a mixture of discrete and continuous variables. Here, we restrict ourselves to the single discrete variable case in order to maximise the size of the datasets used in the experiments, and to ease the interpretation of the effects of confounding on predictions. The models that we now describe, however, can still be applied when there are multiple confounds, and we emphasise this generality by denoting confound variables using the vector c in what follows.

### 4.2 Models used

We use Gaussian Process Regression (GPR) to evaluate the methods in 2, 3 for dealing with biased training samples. Gaussian Processes provide a flexible Bayesian framework for model estimation and they have recently gained popularity for building predictive neuroimaging models for regression and classification (Marquand et al., 2010, Doyle et al., 2013, Young et al., 2013). In our application of GPR, the probabilistic nature of the modelling is only utilized when determining kernel hyperparameters and weights for the Instance Weighting described in Section 3.2: The final predictions are taken as the mean of the resulting posterior distribution which is exactly equivalent to kernel ridge regression, a popular non-Bayesian approach.

Gaussian processes impose a multivariate Gaussian prior on a set of latent variables *f*_*i*_, where the mean and covariance of the prior are functions of the inputs $x_{i}$:

(20) $$\;E(f_{i})=m(x_{i})\;\;\mathrm{Cov}(f_{i},f_{j})=k(x_{i},x_{j}).$$

We assume a zero mean function $m(x_{i})\equiv 0$ throughout this work, as is commonly done (Rasmussen, 2006). The covariance function $k(x_{i},x_{j})$, also referred to as the kernel function, describes how the values of the latent variables covary across the input space, and it has a set of associated kernel parameters θ. The targets *y*_*i*_ are related to the latent variables *f*_*i*_ through the likelihood function. We use the Gaussian Likelihood below for all methods apart from ‘Instance Weighting’:

(21) $$P(y_{i}|f_{i})=\frac{1}{\sigma \sqrt{2\pi }}e^{-\frac{{\left(y_{i}-f_{i}\right)}^{2}}{2\sigma^{2}}}$$

where σ>0 is the standard deviation of the noise. We can thus consider the latent variables *f*_*i*_ to be the unobserved ‘noiseless’ versions of the targets *y*_*i*_, related to the targets via Eq. (21), and with a Gaussian prior distribution defined by Eq. (20). With the above likelihood, the posterior distribution of the target y_*_ of a test point $x_{*}$, given the training data, then has the closed form

(22) $$y_{*}|X,y,x_{*}∼N({\overset{¯}{y}}_{*},\mathrm{Var}(y_{*}))\;\mathrm{where}{\;\overset{¯}{y}}_{*}=k_{*}{\left(K+\sigma^{2}I\right)}^{-1}y\;\;\mathrm{Var}(y_{*})=k(x_{*},x_{*})-k_{*}{\left(K+\sigma^{2}I\right)}^{-1}k_{*}^{T}+\sigma^{2}$$

in which *K* is the *n*×*n* matrix of training set covariances, $K_{\mathrm{ij}}=k(x_{i},x_{j})$, and $k_{*}$ is the n-dimensional row vector of test-training covariances, $k_{*i}=k(x_{*},x_{i})$. We take the predictive function f to be the mean of the posterior ${\overset{¯}{y}}_{*}$ at test point $x_{*}$, i.e.,

(23) $$f(x_{*})=k_{*}{\left(K+\sigma^{2}I\right)}^{-1}y.$$

Note that Eq. (23) is precisely the predictive equation for kernel ridge regression. The values of the parameters θ in the covariance function *k*, and the likelihood parameter *σ*, are estimated by maximising the marginal likelihood Z where Z=P(y∣X,θ,σ) is the probability of the data given the model. The marginal likelihood automatically incorporates a trade-off between model fit and model complexity and so is commonly used to estimate hyperparameters in Bayesian models (Rasmussen, 2006). Maximizing Z is equivalent to maximizing the log marginal likelihood logZ, which for the Gaussian Likelihood is given by Rasmussen (2006)

(24) $$\log Z=-\frac{1}{2}y^{T}{\left(K(\theta )+\sigma^{2}I\right)}^{-1}y-\frac{1}{2}\log\left|K(\theta )+\sigma^{2}I\right|\;-\frac{n}{2}\log2\pi .$$

We now describe our implementation of the standard methods for dealing with confounding described in Section 2 which all use the likelihood and predictive function described above. This is followed by a description of our implementation of the Instance Weighting described in Section 3, which uses a slightly different likelihood and predictive function. For all models, the outputs of the corresponding predictive function $f(x_{*})$ were taken to be the predictions of age for the IXI data, while for the ADNI data, we round $f(x_{*})$ to the nearest whole number within the range of the MMSE score (0-30). In addition, all features were standardized to have zero mean and unit variance using just the training data, before model training.

#### 4.2.1 Images only

The baseline model is one where only the image features are used for prediction, so each input $x_{i}\equiv g_{i}$. In this case, we use a linear kernel plus bias for training and prediction:

(25) $$k(x_{i},x_{j})=\frac{g_{i}g_{j}^{T}}{l^{2}}+b^{2}$$

where the kernel hyperparameters are θ≡(l,b). The use of the above kernel essentially means that the predictive function given in Eq. (23) is a linear model of the image features, which is the most common model used in predictive neuroimaging.

#### 4.2.2 Adjusted images

We produce confounds-adjusted images using Eq. (4), in which both training and test data are used to build the adjustment model and adjusted data is used during training and prediction. GPR training and prediction is then performed using the kernel from Eq. (25), but with inputs $x_{i}\equiv g_{i}^{A}$:

(26) $$k(x_{i},x_{j})=\frac{g_{i}^{A}{g_{j}^{A}}^{T}}{l^{2}}+b^{2}.$$

As for the ‘Images Only’ model, the kernel hyperparameters are θ≡(l,b).

#### 4.2.3 Images & confounds

The confounds are incorporated into the predictive model by appending them to the image features, so that each input to the GPR is $x_{i}\equiv [g_{i},c_{i}]$. We use a kernel that is the sum of the kernel in Eq. (25) and a linear Automatic Relevance Determination (ARD) kernel applied to the confounds only (Rao et al., 2015):

(27) $$k(x_{i},x_{j})=\frac{g_{i}g_{j}^{T}}{l^{2}}+b^{2}+c_{i}\Lambda^{\mathrm{ARD}}c_{j}^{T}$$

where $\Lambda^{\mathrm{ARD}}$ is a diagonal matrix with entries $\frac{1}{l_{1}^{2}},\ldots ,\frac{1}{l_{d_{C}}^{2}}$. The hyperparameters $l_{1}^{2},\ldots ,l_{d_{C}}^{2}$ scale the confounds so that their contribution to the kernel, and hence, the predictive function, is controlled. The kernel hyperparameters for this model are $\theta \equiv (l,b,l_{1},\ldots ,l_{d_{C}})$. The resulting predictive function is then a linear model of the image features and the confounds. Note that the kernel in Eq. (27) is only appropriate for confounds which are continuous or discrete with less than three levels. For discrete confounds with more than two levels, one possible approach is to apply an ARD squared-exponential kernel to a “1-hot” encoding of the discrete confound (Duvenaud, 2014), and add the resulting kernel to that in Eq. (27).

#### 4.2.4 Images only with instance weighting

In order to perform instance weighting, we firstly need to estimate the weights *w*_*i*_

(28) wi=P^S(yi)P^S(yi|ci)

from Eq. (15). This requires estimating the ratio of the densities of *P*(*y*) and P(y|c) from the training sample. In this work, we do this by estimating each of *P*(*y*) and P(y|c) using an independent GP, and dividing them. For *P*(*y*), we use a GP with a kernel consisting only of a bias term:

(29) $$k(x_{i},x_{j})=b^{2}$$,

i.e., θ≡b and the Gaussian Likelihood in Eq. (21). The values of θ,σ are determined by maximising the marginal likelihood in Eq. (24), and then Eq. (22) gives the full posterior for y_*_. Since Eq. (29) does not contain either the image features nor the confounds, this procedure is similar to fitting a normal distribution to the marginal distribution *P*(*y*). We then evaluate the posterior at each *training* point *i* to give the value of P^S(yi). Similarly, we estimate P(y|c) with a GP using an ARD kernel applied only to the confounds, and a bias term:

(30) $$k(x_{i},x_{j})=b^{2}+c_{i\;}\Lambda^{\mathrm{ARD}}c_{j}^{T}$$,

i.e., $\theta \equiv (b,l_{1},\ldots ,l_{d_{C}})$, with a Gaussian Likelihood. Once again, the ARD parameters and the bias are determined by maximising the marginal likelihood. This kernel is the same as the one in Eq. (27) in Section 4.2.3, but without the image features, and so fitting this GP effectively learns a linear relationship between the confounds c and the target variable *y*, in which the contribution of each confound to the kernel is controlled via the estimated ARD parameters. Discrete confounds with a number of levels greater than two can be incorporated into the kernel as described in Section 4.2.3. We can then evaluate the posterior (22) at each training point to give the value of P^S(yi|ci). We can now directly calculate the instance weights for each training example using Eq. (28).

The estimates for the weights in Eq. (15) are clearly dependent on the modelling procedure that is used to determine them. In this work, they were obtained by independently fitting GPs to *P*(*y*) and P(y|c) using the described kernels and Gaussian Likelihood, and taking the ratio of the corresponding posterior densities from Eq. (22), which incorporates uncertainties in the estimated model parameters (which have priors placed on them due to the nature of GPs), given the training data. Whichever method is used, however, it should ideally result in a weighting of the sample to produce a pseudo-sample in which the association between *y* and c has been removed. This observation regarding the property of the weights was noted in Linn et al. (2015), which describes a similar algorithm to the one presented but applied to classification problems, and in the literature pertaining to the estimation of causal effects with continuous treatments (Imai and Ratkovic, 2014). During each application of instance weighting, we therefore check for an improvement in balance of the weighted samples compared to the original samples.

Since in this work, the confound is a discrete variable with two levels (gender for ADNI data, site for IXI data), we do this by calculating the weighted standardized difference (Austin and Stuart, 2015) in the target variable between the two levels of the confound. This is determined as:

(31) $$\frac{m_{Y_{0}}^{\mathrm{Wtd}}-m_{Y_{1}}^{\mathrm{Wtd}}}{\sqrt{\frac{s_{Y_{0}}^{2^{\mathrm{Wtd}}}+s_{Y_{1}}^{2^{\mathrm{Wtd}}}}{2}}}$$

where *Y*_*k*_ refers to the subset of training subjects with level *i* of the discrete confound *c*, and $m_{Y_{k}}^{\mathrm{Wtd}}$, ${s_{Y_{k}}^{2}}^{\mathrm{Wtd}}$ refer to weighted means and weighted sample variances of the target variable over these subjects:

(32) $$m_{Y_{k}}^{\mathrm{Wtd}}=\frac{\sum_{i\in Y_{k}}w_{i}y_{i}}{\sum_{i\in Y_{k}}w_{i}}\;s_{Y_{k}}^{2^{\mathrm{Wtd}}}=\frac{\sum_{i\in Y_{k}}w_{i}}{{\left(\sum_{i\in Y_{k}}w_{i}\right)}^{2}-\sum_{i\in Y_{k}}w_{i}^{2}}\sum_{i\in Y_{k}}w_{i}(y_{i}-m_{Y_{k}}^{\mathrm{Wtd}})$$

Note that Eq. (31) reduces to the usual standardized difference when the weights are equal for all samples. A decrease in the absolute value of the weighted standardized difference after Instance Weighting implies a reduction in the difference between the mean of the target variable y across gender/site, as we would wish.

We now need to perform the weighted prediction in Eq. (17), for which we use the following heterogenous Gaussian Likelihood:

(33) $$P(y_{i}|f_{i})=\frac{1}{\sigma_{i}\sqrt{2\pi }}e^{-\frac{{\left(y_{i}-f_{i}\right)}^{2}}{2\sigma_{i}^{2}}}$$

in which $\sigma_{i}=\frac{\sigma }{\sqrt{w_{i}}}$. It is possible to show that for a test point $x_{*}$ the predictive function $f\equiv {\overset{¯}{y}}_{*}$ for this likelihood is given by

(34) $$f(x_{*})=k_{*}{\left(K+\sigma^{2}W\right)}^{-1}y$$

where W is a diagonal matrix with entries $\frac{1}{w_{i}}$, and K is the kernel used for doing the Instance Weighted predictions. This is then equivalent to a *weighted* kernel ridge regression, in which the loss associated with training point i is weighted by $w_{i}$. We set K using the kernel function in Eq. (25), and estimate kernel hyperparameters θ≡(l,b) and noise parameter σ by maximizing the marginal likelihood for the heterogenous Gaussian Likelihood.

All models were implemented using the GPML toolbox available through http://www.gaussianprocess.org/gpml/code/matlab/doc/. This required determining derivatives of the heterogeneous likelihood in Eq. (33), in order to apply the Instance Weighting. These are given in Appendix B.

### 4.3 Evaluation methodology

The first aim of our experiments is to assess how well the methods described in 2, 3 perform in the presence of confounding, in terms of predictive accuracy. This requires us to train each model using biased training samples, but test them on unbiased samples that are representative of the population-of-interest. Cross-validation is therefore an inappropriate scheme for evaluating models in the presence of confounding, since in that case the test sample is the same as the training sample, and so cannot be an unbiased sample. Although this point is mentioned in Kostro et al. (2014), where it was noted that using cross-validation may give misleadingly high predictive accuracies if confounds are included as predictors in the model, it is important to appreciate that the standard application of cross-validation is, in general, inappropriate for evaluating predictive accuracy when using methods that attempt to control for confounding: The test set must be an unbiased sample, representative of the population-of-interest, in order to assess the predictive accuracies of different approaches.

The second aim of our experiments is to assess the impact of confounding on the predictions, and so we perform an additional analysis without confounding in which we use the same unbiased test samples but train with unbiased training samples that are representative of the population-of-interest. This enables us to not only compare predictive accuracies with and without confounding, but also to assess the impact of confounding on the distribution of predictive accuracies across the population-of-interest.

#### 4.3.1 ADNI validation scheme

**Fig. 2:**
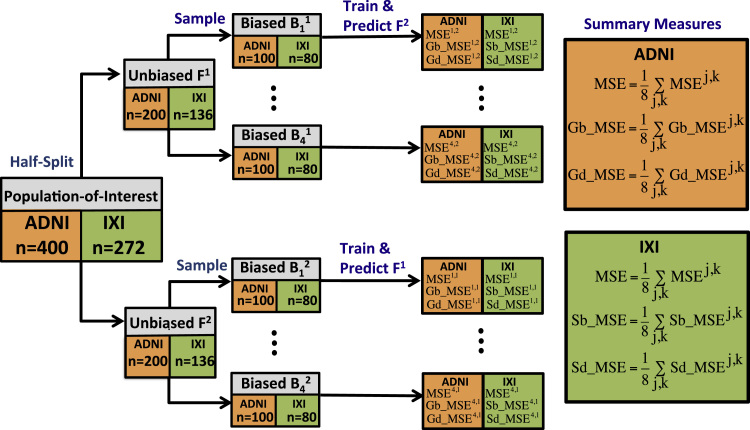
Schematic for the evaluation of each model using ADNI and IXI data. The sizes of each sample and evaluation measures are shown in orange in for the ADNI data, and green for the IXI data. The figure shows the procedure when testing the models in the presence of confounding. The same procedure is used for testing the models when there is no confounding, but there the samples $B_{j}^{k}$ are unbiased.

The orange squares in Fig. 2 give the overall schematic for the evaluation with ADNI data, which we now describe.

**Fig. 3:**
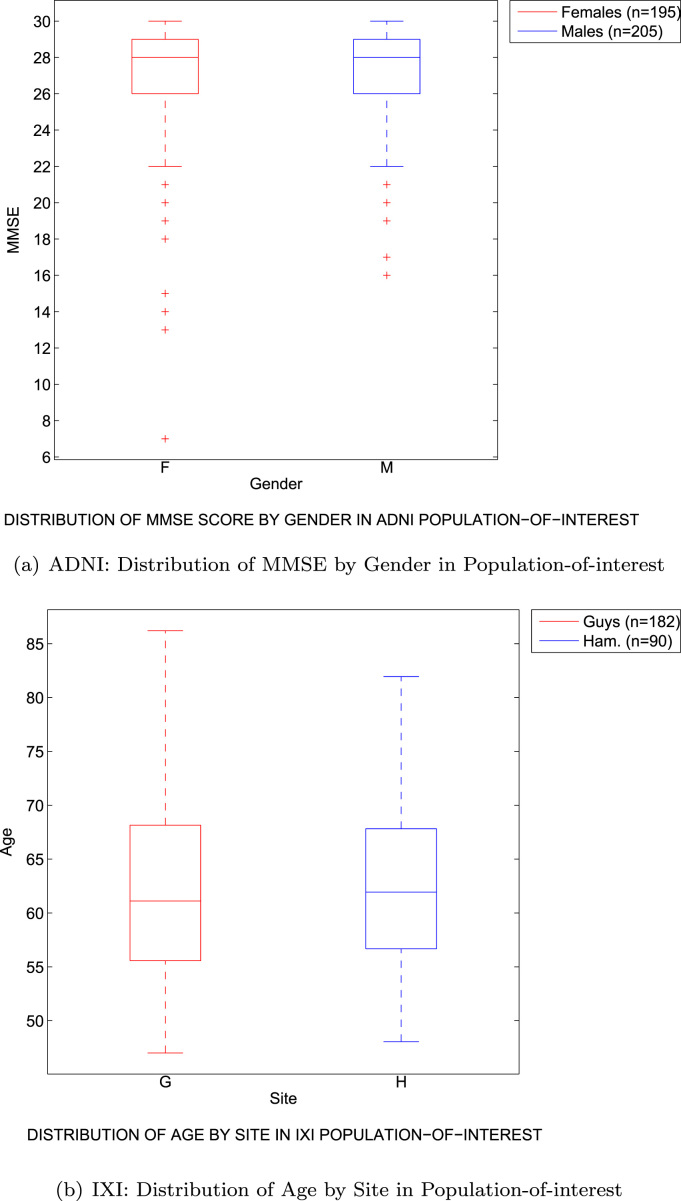
This figure shows the distribution of the target variables by confound for the ADNI data (a) and IXI data (b). For the ADNI data, there is no significant difference between the MMSE scores of each gender, and there is approximately the same number of females and males in the 400 subjects. In the IXI data, there is no significant difference between the ages of subjects from each site, and there is approximately twice as many subjects from Guys Hospital as there are from Hammersmith Hospital in the 272 subjects. The overall means, standard deviations of the target variables in each dataset are ADNI (MMSE): 27.07, 3.32 and IXI (age): 62.35, 8.14.

We firstly produced our ‘population-of-interest’ by selecting 400 subjects from the original 592 subjects in which gender is not significantly associated with the MMSE score (2-sided Student's t-test, p=0.45). Fig. 3(a) shows the distribution of the MMSE score for each gender over the 400 subjects. These subjects are then half-split into 2 folds of data $F^{1},F^{2}$ each of which is an unbiased sample of size 200 from the population-of-interest.

**Fig. 4:**
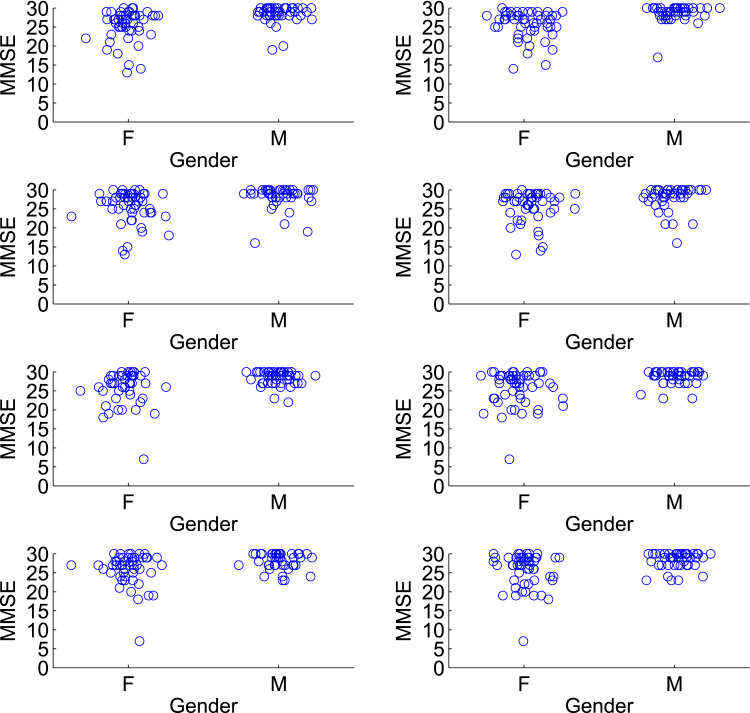
This figure shows the distribution of the MMSE score by gender for each of the biased training samples drawn from the ADNI data. In these samples, males tend to have a higher MMSE score than females.

For model evaluation under confounding, we sample 4 biased training sets $B_{1}^{1},\ldots ,B_{4}^{1}$ of size 100 from $F^{1}$, each with significant associations between gender and MMSE (2-sided Student's t-test, p<0.05), such that being male is associated with a higher MMSE score. These samples, shown in Fig. 4, are produced by sampling from $F^{1}$ non-uniformly according to a model in which males are more likely to be chosen than females as the MMSE score increases. For a given model, we train using each $B_{j}^{1}$ and predict the unbiased sample $F^{2}$, giving four sets of predictions {y^ij,2} where i indexes over the subjects in $F^{2}$, and j=1,…,4 indexes the biased training sample. This procedure is repeated after switching the roles of $F^{1}$ and $F^{2}$, giving four more sets of predictions {y^ij,1} where i indexes over the subjects in $F^{1}$, and j=1,…,4 refers to a biased training sample $B_{j}^{2}$ drawn from $F^{2}$. For evaluating predictive performance when there is no confounding, we repeat the whole procedure but here each of the 8 samples $B_{j}^{k}$ is an unbiased sample from $F^{k}$, in which there is no significant association between MMSE score and gender. The unbiased samples are produced by uniform random sampling of each fold $F^{k}$.

#### 4.3.2 IXI validation scheme

**Fig. 5:**
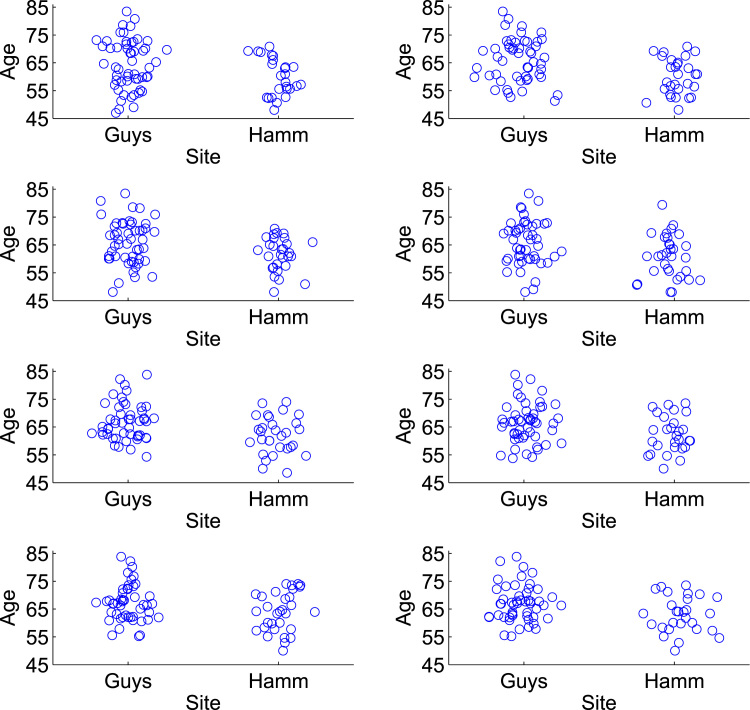
This figure shows the distribution of age by site for each of the biased training samples drawn from the IXI data. In these samples, subjects from Guys tend to be older than subjects from Hammersmith.

The evaluation for the IXI data proceeds in an analagous manner to that for the ADNI data, and is shown in the green squares of Fig. 2. Here, the initial pool of 274 subjects already contains no association between site and the age of the subjects, but we remove a subject with a poor segmentation and randomly remove one more subject to given an even size for the population-of-interest. Site is not significantly associated with age in the resulting 272 subjects (2-sided Student's t-test, p=0.77), and Fig. 3(b) shows the distribution of age for each site over the 272 subjects. The two folds of data $F^{1},F^{2}$ are now of size 136, and the samples $B_{j}^{k}$ are of size 80. For the experiment in the presence of confounding, the samples $B_{j}^{k}$ are biased and contain a significant association between site and age (2-sided Student's t-test, p<0.05), with subjects from Guy's tending to be older than those from Hammersmith's Hospital. These samples, shown in Fig. 5, are produced by creating a linear relationship between site and age (considering site as a continuous variable), and then sampling from each $F^{k}$ non-uniformly to prefer subjects that fit this relationship. The corresponding samples for the experiment without confounding do not have a significant association between site and age, and these are produced by uniform random sampling from each $F^{k}$. Note that in the population-of-interest, the ratio of the number of subjects from Guy's Hospital to the number from Hammersmith Hospital is approximately 2:1. We approximately preserve this ratio in our samples, with a geometric mean ratio of 1.75:1 in the biased samples, and 1.89:1 in the unbiased samples.

#### 4.3.3 Prediction metrics

For each dataset and each analysis, we calculate a number of different metrics using the 8 sets of predictions y^ij,k. Firstly we determine the mean-squared-error (MSE) for each of the 8 sets:

(35) MSEj,k=1|Fk|∑i∈Fk(yi−yi^j,k)2

where ${\mathrm{MSE}}^{j,k}$ is the MSE when predicting fold $F^{k}$ using the j th training sample, and $\left|F^{k}\right|$ is the size of the fold. The 8 MSE values are then averaged to give MSE which summarizes the predictive accuracy of a model when predicting unbiased samples from either biased samples (evaluation under confounding), or unbiased samples (evaluation without confounding.)

The simulated example in Appendix A shows that bias in the training sample can alter how the prediction errors vary across the population-of-interest with respect to the values of the confound and the target variable. We therefore calculate additional measures to explore this phenomenon with both the ADNI and IXI experiments. For the ADNI data, we do this by partitioning each of the folds $F^{k}$ into 2 subsets $R_{1}^{k},R_{2}^{k}$ by MMSE score:

(36) $$R_{1}^{k}=\left\{i\in F^{k}:y_{i}<=28\right\}\;R_{2}^{k}=\left\{i\in F^{k}:29<=y_{i}<=30\right\}.$$

We choose 28 as the partition threshold because it is the median of the MMSE scores of the 400 subjects, and Table 1 shows the number of females/males within each $R_{l}^{k}$. We also define the set of females/males in each fold $F^{k}$ to be $C_{0}^{k}$ and $C_{1}^{k}$ respectively. The gender-balanced test errors for each subset $R_{l}^{k}$ when using training sample j are calculated as

(37) Gb_MSElj,k=(12∑q=011nqlk∑i∈Rlk∩Cqk(yi−yi^j,k)2)

where $n_{\mathrm{qlk}}$ is the number of subjects in subset $R_{l}^{k}$ with gender q. The measures $\mathrm{Gb}\_{\mathrm{MSE}}_{l}^{j,k}$ are summarized by the number $\mathrm{Gb}\_{\mathrm{MSE}}^{j,k}$:

(38) $$\mathrm{Gb}\_{\mathrm{MSE}}^{j,k}=\frac{1}{200}\overset{2}{\underset{l=1}{\sum }}n_{\mathrm{lk}}\times \mathrm{Gb}\_{\mathrm{MSE}}_{l}^{j,k}$$

where $n_{\mathrm{lk}}$ is the number of subjects in subset $R_{l}^{k}$. This quantity is the weighted average of the gender-balanced test errors for each subset of subjects $R_{l}^{k}$, where the weights are the size of each subset. We average $\mathrm{Gb}\_{\mathrm{MSE}}^{j,k}$ over all 8 sets to give the overall gender-balanced error Gb_MSE. In addition to the gender-balanced errors, we determine the difference in the errors for males and females over the MMSE scores. Unlike MSE and Gb_MSE, these gender-difference errors do not measure prediction accuracy, but instead enable us to assess whether one gender is being predicted better than the other as the value of the MMSE score changes. We define the signed gender-difference error for each subset $R_{l}^{k}$ when using training sample j as the signed difference between the MSE for females and males:

(39) Sg_Gd_MSElj,k=1n0lk∑i∈Rlk∩C0k(yi−yi^j,k)2−1n1lk∑i∈Rlk∩C1k(yi−yi^j,k)2.

The measures $\mathrm{Sg}\_\mathrm{Gd}\_{\mathrm{MSE}}_{l}^{j,k}$ are summarized by the weighted average of their absolute values

(40) $$\mathrm{Gd}\_{\mathrm{MSE}}^{j,k}=\frac{1}{200}\overset{2}{\underset{l=1}{\sum }}n_{\mathrm{lk}}\times \left|\mathrm{Sg}\_\mathrm{Gd}\_{\mathrm{MSE}}_{l}^{j,k}\right|$$

where the absolute value is used to prevent positive and negative values cancelling each other in the weighted sum. We then average $\mathrm{Gd}\_{\mathrm{MSE}}^{j,k}$ over all 8 sets to give the overall gender-difference error Gd_MSE, which describes the magnitude of the difference between how well each gender is predicted over the range of the MMSE score.

We determine corresponding measures for the IXI data by partitioning each of the folds $F^{k}$ into 2 subsets by age:

(41) $$R_{1}^{k}=\left\{i\in F^{k}:y_{i}<=61.54\right\}\;R_{2}^{k}=\left\{i\in F^{k}:y_{i}>61.54\right\}$$

where the threshold of 61.54 is the median of the ages of the 272 subjects. The number of subjects from each site within each of the subsets $R_{l}^{k}$ for each test fold is given in Table 2. Site-balanced errors $\mathrm{Sb}\_{\mathrm{MSE}}^{j,k}$ are given by

(42) $$\mathrm{Sb}\_{\mathrm{MSE}}^{j,k}=\frac{1}{136}\overset{2}{\underset{l=1}{\sum }}n_{\mathrm{lk}}\times \mathrm{Sb}\_{\mathrm{MSE}}_{l}^{j,k}$$

where $n_{\mathrm{lk}}$ is the number of subjects in subset $R_{l}^{k}$, and $\mathrm{Sb}\_{\mathrm{MSE}}_{l}^{j,k}$ is calculated in the same way as $\mathrm{Gb}\_{\mathrm{MSE}}_{l}^{j,k}$, but with q referring to site instead of gender in Eq. (37). The overall site-balanced error Sb_MSE is then determined by averaging $\mathrm{Sb}\_{\mathrm{MSE}}^{j,k}$ over the 8 datasets. Site-difference errors $\mathrm{Sd}\_{\mathrm{MSE}}^{j,k}$ are given by

(43) $$\mathrm{Sd}\_{\mathrm{MSE}}^{j,k}=\frac{1}{136}\overset{2}{\underset{l=1}{\sum }}n_{\mathrm{lk}}\times \left|\mathrm{Sg}\_\mathrm{Sd}\_{\mathrm{MSE}}_{l}^{j,k}\right|$$

where $\mathrm{Sg}\_\mathrm{Sd}\_{\mathrm{MSE}}_{l}^{j,k}$ is calculated in the same way as $\mathrm{Sg}\_\mathrm{Gd}\_{\mathrm{MSE}}_{l}^{j,k}$, but with q referring to site instead of gender in Eq. (40). The overall site-difference error Sd_MSE is determined by averaging $\mathrm{Sd}\_{\mathrm{MSE}}^{j,k}$ over the 8 datasets.

#### 4.3.4 Significance testing

Permutation tests are used to determine whether the predictive performance of the models, as measured by MSE, Gb_MSE (ADNI data) and Sb_MSE (IXI data), are significantly better than chance. These are performed by calculating MSE, Gb_MSE and Sb_MSE after training with the values of the targets in the 8 training/test pairs randomly shuffled. During the shuffling, we ensure that the target values in both the training data and the test data are permuted within gender for the ADNI data, while for the IXI data they are permuted within site. The motivation for this relabelling scheme is that it preserves the confounding association between the targets and the confound, while breaking the relationship between the imaging data and the targets. Such a permutation test is an example of one with restricted permutations (Good, 2005), where the relabellings ensure that the targets are exchangeable under the null hypothesis of there being no relationship between the targets and the imaging data, given the confound, i.e., the targets are conditionally independent of the imaging data given the confound. This modification to the standard permutation scheme used when assessing predictive models in neuroimaging enables us to test whether, in the presence of confounding, the predictive model is learning ‘real’ information that is useful for predicting the target rather than information that is associated with the confound. To the best of our knowledge, we have not seen such a permutation test proposed for assessing predictive performance in the presence of confounding. We perform five hundred (including the true targets) permutations and count how many times the models give metrics that are less than or equal to the metrics with the true targets. This number is then divided by 500 to give a p-value for whether the model is learning real predictive information from the imaging data.

### 4.4 Results with ADNI data

#### 4.4.1 Prediction errors

**Fig. 6:**
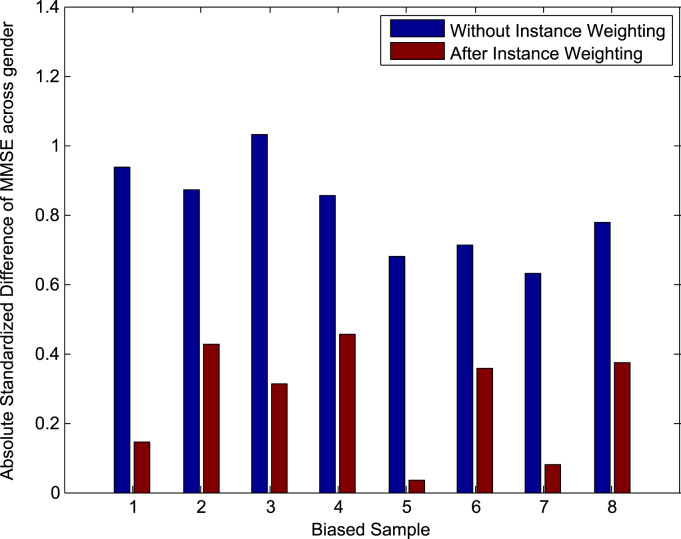
Absolute standardized difference of MMSE score across gender in each of the eight biased samples. The blue bars show the values in the original samples, while the brown bars show the values using the weighted samples calculated according to Eq. (31).

Table 3(a) gives the measures of predictive accuracy, MSE and Gb_MSE, for the different models using biased training samples. We can see that all models perform better than chance and so the models are able to learn information that is useful for prediction of the unbiased data despite the presence of confounding. Instance weighting does not appear to have improved predictive accuracy compared to using the original images and, in fact, gives identical sets of predictions in 3 of the 8 samples to those of the baseline model. However, we do see a reduction in the absolute value of the weighted standardized difference of the MMSE score across gender in the biased samples after Instance Weighting, as shown in Fig. 6. This indicates that the reweighting of subjects in the biased samples according to the Instance Weights produces a more balanced pseudo-sample for training. Using adjusted images shows a small improvement with respect to MSE but a small degradation with respect to Gb_MSE. The ‘Images & Confounds’ model gives much worse predictions than all the other models: The combination of covariate shift and model misspecification appears to have affected this model particularly badly. The corresponding measures using unbiased training samples are shown in Table 3(b) and once again, all models perform better than chance. We find that both MSE and Gb_MSE improve for all models, including the baseline ‘Images Only’ model, when using unbiased rather than biased training samples. This suggests that it is important to use unbiased training samples when building predictive models in order for them to perform optimally on the population-of-interest.

#### 4.4.2 Gender-difference & signed gender-difference errors

Table 4 shows the summary gender-difference measure Gd_MSE for the different models. We can see that for the baseline ‘Images Only’ model, this measure is similar whether the training samples are biased or unbiased. The same is true for the ‘Images & Confounds’ model, while the adjusted images model gives relatively high Gd_MSE measures when the training sample is biased. Conversely, the ‘Instance Weighted’ model gives a similar Gd_MSE measure to the baseline model with unbiased samples, but a reduced measure when the training sample is biased.

**Fig. 7:**
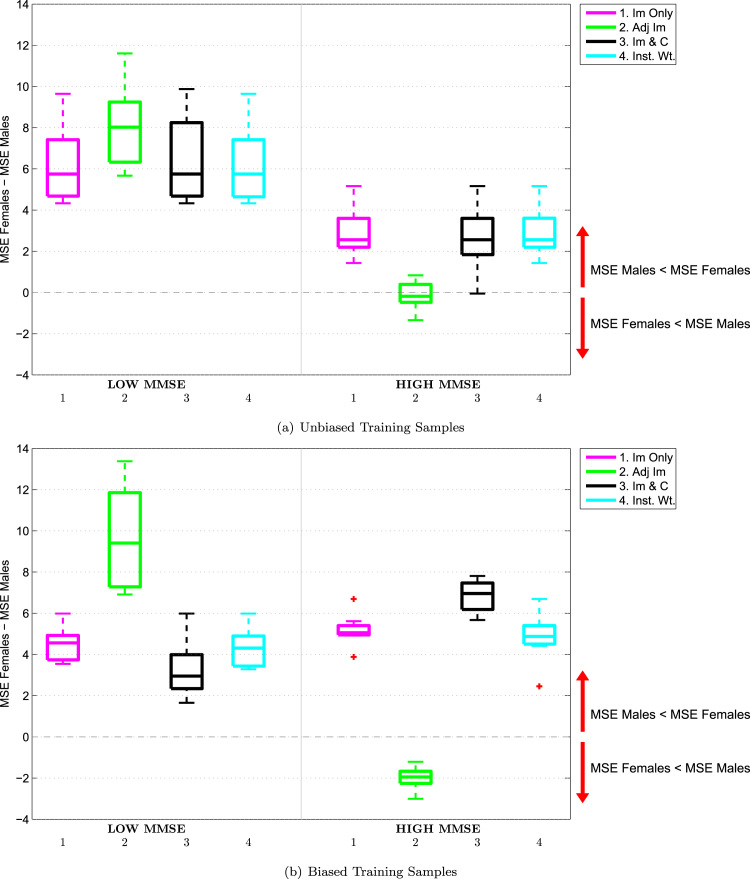
Signed difference between the MSE for females and MSE for males, over the different ranges of the MMSE Score. The data points in each box plot are the 8 predictions performed for a particular model. Results using unbiased training samples are shown in (a), while results using biased training samples are shown in (b). In the biased samples, males tend to have higher MMSE scores.

The impact of confounding on the difference between the prediction accuracies for each gender can be further analysed by examining the boxplots in Fig. 7. Here, each boxplot shows the signed gender difference errors for low MMSE scores on the left, $\mathrm{Sg}\_\mathrm{Gd}\_{\mathrm{MSE}}_{1}^{j,k}$, and the corresponding metric for high MMSE scores on the right, $\mathrm{Sg}\_\mathrm{Gd}\_{\mathrm{MSE}}_{2}^{j,k}$. (Note that, for clarity of exposition, Fig. 7 presents the results using unbiased training samples before those using biased samples.).

If we firstly consider the results for the baseline ‘Images Only’ model (shown in pink) when using the unbiased training samples, shown in Fig. 7(a), we can see how predictive accuracies vary across gender and MMSE score: For subjects with a low MMSE score, males appear to be much better predicted than females, while for subjects with a high MMSE score, males are still better predicted but to a lesser degree. We can see the impact of confounding on the distribution of prediction errors if we now consider the corresponding results using biased training samples in Fig. 7(b). Recall that in the biased training samples, being male is associated with a higher MMSE score than being female. For the ‘Images Only’ model, we can see that for subjects with low MMSE scores, the signed difference between the MSE for females and that for males *decreases* compared to the corresponding result in Fig. 7(a), i.e., the predictions move in a direction that favours the prediction of females rather than males. This corresponds with the bias in the training samples, where females tend to have lower MMSE scores. Conversely, for subjects with high MMSE scores, the signed difference between the MSE for females and males *increases* compared to the corresponding results with the unbiased training samples, so the predictions move in a direction that favours the prediction of males rather than females. This again corresponds with the bias in the training samples, where males tend to have higher MMSE scores. The bias in the training samples therefore causes a shift in the difference between how well each gender is predicted over the range of targets, in the direction of this bias. Note that this shift occurs even though gender has not been included as a feature in this model. A similar shift is seen with the ‘Instance Weighted’ model, while the shift is amplified with the ‘Images & Confounds’ model, again demonstrating that including a confound in a predictive model can potentially have undesirable effects.

Lastly, we consider the impact of confounding on the prediction accuracies for the ‘Adjusted Images’ model by comparing the boxplots in Fig. 7. In contrast to the other approaches, we find that bias in the training samples causes shifts in the difference between how well each gender is predicted over the range of targets in the *opposite* direction to the bias in the training sample: For subjects with low MMSE, the signed difference between the MSE for females and males *increases* when using biased training samples, while for subjects with high MMSE, the corresponding measure *decreases*. One may therefore consider image adjustment to have in some sense ‘overcompensated’ for the bias during model training.

#### 4.4.3 Weight vectors

**Fig. 8:**
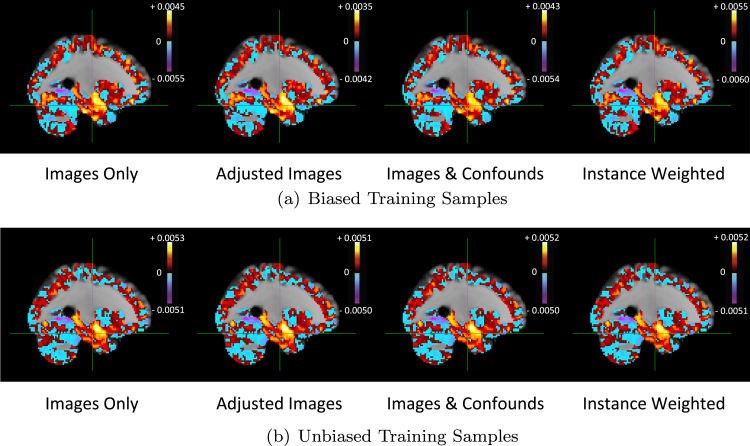
This figure shows the average weight images for each model when training with biased and unbiased samples from the ADNI data. Positive weights are indicated with a hot colour and negative weights with cool colours.

For illustration, Fig. 8(a) shows the average weight vectors for each model when using the biased training sample, with hot colours indicating positive weights and cool colours indicating negative weights. A positive/negative weight at a voxel v indicates an increase/decrease in the predictions of the target as the value of the image feature at v increases, holding the values of all other features constant. The weight vectors for the approaches are not substantially different and all indicate large positive weights in the left hippocampus/amygdala which are proximal to the crosshair, positioned at (−26,−14,−22) in MNI space. Fig. 8(b) shows the average weight vectors for each model when using the unbiased training sample. The weight vectors for the approaches are once again similar.

### 4.5 Results with IXI data

#### 4.5.1 Prediction errors

**Fig. 9:**
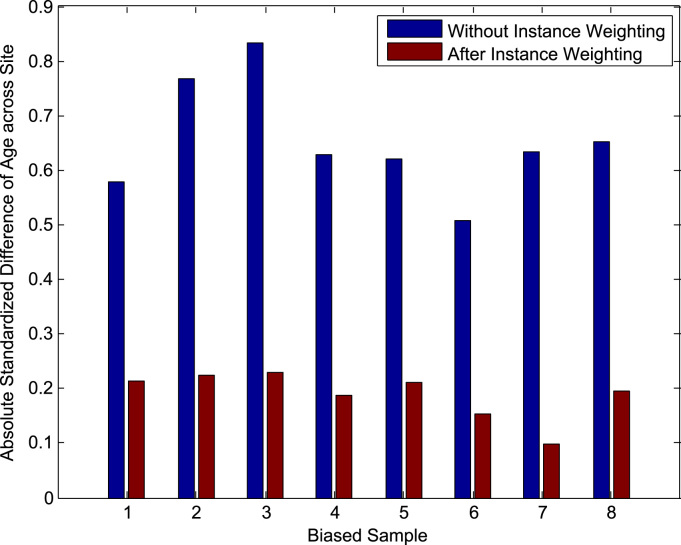
Absolute standardized difference of age across site in each of the eight biased samples. The blue bars show the values in the original samples, while the brown bars show the values using the weighted samples calculated according to Eq. (31).

Table 5(a) gives the error measures for the different models using biased training samples. All models perform better than chance according to MSE and Sb_MSE and so the models are able to learn information that is useful for prediction of the unbiased data despite the presence of confounding. The baseline and ‘Instance Weighted’ models give very similar predictions over the 8 samples. In fact, over 6 of the samples the predictions are identical, while for 2 of the samples the ‘Instance Weighted’ models give a small improvement, producing the slight reduction in MSE and Sb_MSE for this approach. We do, however, see a reduction in the absolute value of the weighted standardized difference of age across site in the biased samples after Instance Weighting, as shown in Fig. 9. which indicates that the Instance Weighting is producing a more balanced pseudo-sample for training. As with the ADNI data, the ‘Images & Confounds’ model performs worse than the baseline model due to the combination of covariate shift and model misspecification. The ‘Adjusted Images’ model performs worst of all, indicating that the adjustment procedure has not been able to transform the biased samples into unbiased samples. This indicates that the simple linear model used to remove variability in the image data associated with the confound may not appropriate, but in practice we will not know the correct form of model to perform the adjustment. The corresponding measures using unbiased training samples are shown in Table 5(b) and once again, all models perform better than chance. As with the ADNI data, all models perform better when training with the unbiased samples compared to when using the biased sample according to the accuracy measures MSE and Sb_MSE, providing further evidence of the importance of training with unbiased samples in predictive modelling.

#### 4.5.2 Site-difference and signed site-difference errors

Table 6 shows the site-difference errors for the IXI data. We can see that the summary measure Sd_MSE reduces somewhat for the ‘Images Only’ and Instance Weighting models, when using the biased training samples compared to when using the unbiased samples. It increases slightly for the ‘Images & Confounds’ model when using biased training samples, while the adjusted images model gives a large increase in Sd_MSE when the training sample is biased.

**Fig. 10:**
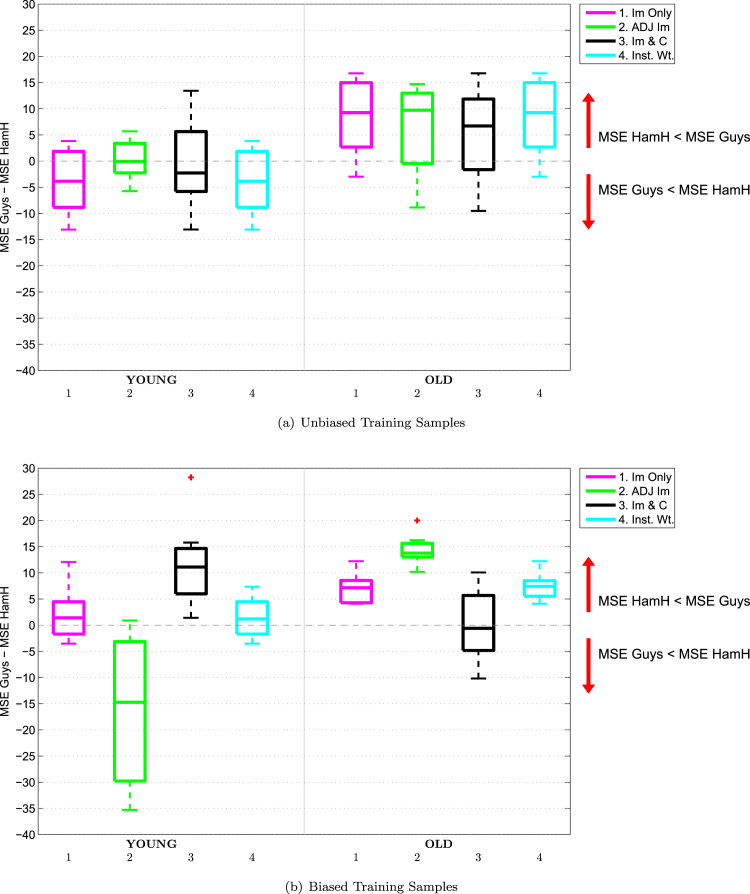
Signed difference between the MSE for Guys and Hammersmith subjects, over the different ranges of age. The data points in each box plot are the 8 predictions performed for a particular model. Results using unbiased training samples are shown in (a), while results using biased training samples are shown in (b). In the biased samples, subjects from Guys tend to be older.

We can further analyse the impact of confounding on the difference between the prediction accuracies for each site by examining Fig. 10, in which each boxplot shows the signed site difference errors for younger subjects on the left, $\mathrm{Sg}\_\mathrm{Sd}\_{\mathrm{MSE}}_{1}^{j,k}$, and older subjects on the right, $\mathrm{Sg}\_\mathrm{Sd}\_{\mathrm{MSE}}_{2}^{j,k}$. Considering firstly the baseline ‘Images Only’ model when using unbiased training samples, shown in Fig. 10(a), subjects acquired at Guys Hospital are predicted better than those from Hammersmith for the younger subjects, while the reverse is true for the older subjects. We now consider the results using biased training samples in Fig. 10(b), in which subjects acquired from Guys tend to be older than those acquired at Hammersmith. For the ‘Images Only’ model, we can see that for younger subjects, the signed difference between the MSE for Guys and Hammersmith subjects *increases* compared to the corresponding result in Fig. 10(a), i.e., the predictions move in a direction that favours the prediction of subjects from Hammersmith over those from Guys. This corresponds with the bias in the samples, where subjects from Hammersmith tend to be younger than those from Guys. Conversely, for older subjects, the signed difference between MSE for Guys and Hammersmith subjects *decreases* compared to the corresponding results with the unbiased sample, i.e., the predictions move in a direction that favours the prediction of subjects from Guys over those from Hammersmith. Once again this corresponds with the bias in the samples, where subjects from Guys tend to be older than those from Hammersmith. As with the ADNI data, the bias in the training sample has caused a corresponding shift in the distribution of prediction errors in the direction of this bias, even though site was not included in the model. This shift is amplified with the ‘Images & Confounds’ model, and is very slightly reduced with the ‘Instance Weighted’ model. This indicates that, even though the Instance Weighted model only improves on the baseline ‘Images Only’ model for 2 of the biased training samples, the improvements in prediction accuracy are in the opposing direction to the bias in the the training sample.

Interestingly, the adjustment model once again does not follow the trend of the other models: For younger subjects, the signed difference between the MSE for Guys and Hammersmith subjects *decreases* when using the biased samples, while for older subjects, the corresponding measure *increases* rather than decreases. We may interpret these results in a similar fashion to those with the ADNI data, i.e., image adjustment tends to overcompensate for bias in the training samples.

#### 4.5.3 Weight vectors

**Fig. 11:**
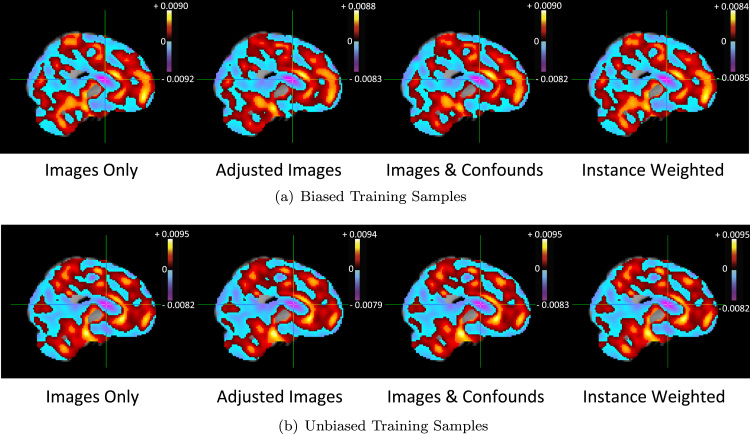
This figure shows the average weight images for each model when training with biased and unbiased samples from the IXI data. Positive weights are indicated with a hot colour and negative weights with cool colours.

Fig. 11(a) shows the average weight vectors for each model when using the biased training sample, with the cross-hair positioned at (12,4,14) in MNI space. Hot colours indicate positive weights and cool colours indicate negative weights. The weight vectors for the approaches are not substantially different and all indicate large negative weights in the right caudate which are proximal to the crosshair. Fig. 11(b) shows the average weight vectors for each model when using the unbiased training sample. The weight vectors for the approaches are once again similar.

### 4.6 Supplementary experiments

Although the main focus of this work is predictive modelling in the presence of confounding using high dimensional voxel-based features, we also repeated our evaluation of the different models using low dimensional region-of-interest (ROI)-based features. This allows us to further investigate the attributes of the different approaches when dealing with confounding, and full details of these experiments are given in Appendix C. Note that the dimensionality of the ROI-based features was 116, which is greater than the size of the training samples in both datasets. This contrasts with Linn et al. (2015), where the size of the training samples was required to be greater than the dimensionality of the ROI-based features, due to the limitations of the particular algorithm used.

## 5 Discussion and conclusions

In this paper, we have discussed and evaluated different approaches for dealing with confounding in the context of predictive modelling in neuroimaging. We began by introducing the concepts of biased and unbiased samples and giving a working definition for confound in the context of predictive modelling in Section 1. While the definition of confound was quite general, our focus in this work was on the specific case of confounding where the data samples were biased as they contained an association between confound and target while they are independent in the population-of-interest. Standard methods for dealing with confounding such as image adjustment were described and we illustrated the consequences of confounding by use of a synthetic example in Appendix A. An instance weighting scheme for dealing with confounds was described in Section 3, and we then performed a thorough evaluation of Instance Weighting and standard methods for dealing with confounding in Section 4 using imaging data from the ADNI and IXI databases. We found that when training with biased samples, the predictive performance of the models when applied to the population-of-interest was lower than when training with unbiased samples. In addition, the bias in the training samples caused a shift in the prediction errors in the direction of the bias for all models apart from image adjustment, for which the prediction errors were shifted in the opposite direction to the bias. Lastly, we found that none of the methods for dealing with confounding gave more accurate predictions than the baseline ‘Images Only’ model for both datasets, although including the confound as a predictor gave models that were less accurate than the baseline model in each case. We now discuss several concerns raised by our evaluation that are relevant to building and assessing predictive models in neuroimaging that we wish to take into clinical practice, before considering other types of confounding that fall within our definition but were not the focus of this particular study. We conclude with an illustrative example.

### 5.1 Impact of bias on predictions

Firstly, we have shown the importance of using samples that are unbiased, with respect to our population-of-interest, for training our predictive models. In practice, this means we should strive to acquire data in which the distribution of potential confounds and clinical groups/variables that we aim to predict, are as close as possible to the population-of-interest over which we intend to apply the predictive model. If we do not, we may find that not only does our overall predictive accuracy degrade, but also that our model may favourably predict certain strata of subjects e.g., female subjects within a particular clinical group, over others. Although, in our experiments, we found that the favourable prediction of certain strata can occur even if the training sample is unbiased, we also found that bias appeared to modify this distribution according to the relationship between the confound and the target in the biased sample. As we have seen, these effects of bias on model training can occur even if we do not explicitly include the confound as a predictor in the model.

### 5.2 Accounting for bias during model training

If we have already acquired a biased sample and wish to train a predictive model, then we can either discard subjects in order to create a matched sample, or use a method that attempts to deal with bias in the sample so that all data can be used during model training. In our evaluation, we considered three such methods: Image Adjustment, incorporating the confounds as predictors, and Instance Weighting. Considered over both the ADNI and the IXI datasets, we found that in the case of learning predictive models from high-dimensional features in the presence of confounding, none of the methods for dealing with confounds performed appreciably better than the others. Instance Weighting, while well motivated, did not appear to improve predictive performance for either the ADNI nor IXI datasets compared to the baseline model. Image adjustment gave slightly better predictions than the ‘Images Only model’ for the ADNI dataset, but the worst predictions of all the models for the IXI dataset. In addition, image adjustment appeared to increase the difference between the prediction errors for each gender for the ADNI dataset, and each site for the IXI dataset, when considered over subranges of the predicted target. The strongest result from our evaluation regarding the different approaches was that including confounds as predictors gave worse predictions than the baseline model in all of the experiments. As was described in Section 3 and shown by the simulated example in Appendix A, the combination of model misspecification and a biased training sample causes predictions to degrade for unbiased samples that are representative of the population-of-interest. Although, in practice, any model is bound to be misspecified, a model in which we explicitly include the confounding variable as an input feature may be prone to overfit the confound to the target in the presence of bias. The degree to which this changes predictive accuracy will most likely depend on how exactly the confounds are included as predictors, but due to the exploratory, data-driven nature of predictive modelling in neuroimaging, this choice is non-trivial.

Whilst we were unable to show a consistent improvement from using either image adjustment or Instance Weighting in our experiments, it is worth discussing the advantages and disadvantages of each of these approaches. If we know the ‘correct’ adjustment model then image adjustment is attractive, since it enables us to remove confounding by transforming the image data into new data that can be considered as having been produced by subjects with identical values of the confounds. Reduction in the variability of the image data associated with the confound may also enable more accurate predictions. Determining the correct adjustment model, however, may be problematic, and if we estimate a bad model this may result in a dataset with greater, rather than less, bias than the original imaging data. It is possible that this is why the accuracies for this approach reduced when applied to the IXI data. In contrast, Instance Weighting essentially aims to weight the examples in such a way as to simulate an unbiased sample so we no longer have to determine an adjustment model. However, now we require estimates for the ratio of the marginal density of the target, P^(y), to the conditional density of the target given the confound, P^(y|c), in order to give the instance weights. Although these densities tend to be quite low-dimensional due to the relatively small number of confounds, the calculation of the weights still presents a potential source of instability due to the division of the two estimated probability densities. Considered over the ADNI and IXI datasets, the weighting of examples gave different predictions to the unweighted ‘Images Only’ model in 7 out of the 16 training samples, and the ratios of the largest to smallest weight in each Instance Weighted model were between 2 and 8 apart from one model for which the ratio was 31. While the weighting of examples does therefore impact the predictive models, it is possible that the extremely high dimensionality of features attentuates the influence of the weighting, preventing the resulting models from having a high variance which is often the case when using weighting approaches (Shimodaira, 2000). Further applications of weighting approaches to different datasets across different feature dimensionalities may provide additional insight as to the nature of this attenuation. In addition, it is worth noting that various approaches to modifying or constraining the estimated weights have been proposed in the context of estimating causal effects (Cole and Hernán, 2008, Imai and Ratkovic, 2014), and it may be interesting to also explore their application to neuroimaging data in further work. Further work investigating the effectiveness of the different methods presented for dealing with confounding should also explore more complicated confounding relationships than that presented here. A method that deals with confounding should ideally be effective when there are strongly non-linear relationships between the confound and the imaging data and/or the confound and the target variable. In this study, we did not actively impose non-linearity on these relationships, but in practice their nature will depend on the particular dataset under consideration.

### 5.3 Model evaluation in the presence of bias

A key aspect of this study is our particular experimental set-up in which the training sample is biased but the test sample is unbiased, which contrasts with previous works (Dukart et al., 2011, Kostro et al., 2014). This allowed us to assess both qualitatively and quantitavely, the effect of bias on prediction errors in the population-of-interest. This is important if we want to estimate how well a model will perform in clinical practice. Alternative evaluation paradigms, such as cross validation using a single biased sample, may give misleading estimates of predictive accuracies for models, because now the test sample and the training sample are the same and so share the same bias. In other words, cross-validation implicitly assumes that the training sample is representative of the population-of-interest, which may not be the case. One should therefore be extremely careful when using cross-validation to evaluate models in the presence of confounding, as the obtained predictive accuracies may lead to false conclusions. In addition, we also described how permutation tests can be modified if we wish to test whether a model is able to learn predictive information in the presence of confounding that is beyond that due to the relationship between the confound and the target.

Of course, in practice, we will not know if our data sample is biased or not, because we can only check for bias by looking at relationships between the target variable and potential confounds which we have actually acquired. Due to the small sample size of imaging studies, there will almost certainly be so-called ‘hidden’ confounds, i.e., variables that we did not acquire for our subjects, that affect the image data and whose relationship with the target variable differs from that in the population-of-interest. These would then have the same adverse effects on model evaluation as known confounds which we do not control for. In our experiments, we ignored any extra possible confounding effects of variables such as age, in the prediction of MMSE score (ADNI), and gender in the prediction of age (IXI). In fact, for the IXI data, gender was not significantly associated with age over the complete set of 272 subjects nor for any of the biased or unbiased samples, and so cannot be considered a hidden confound. In the ADNI data, age was significantly correlated with MMSE over the set of 400 subjects (Pearson's Correlation, p<0.05), and significantly correlated with MMSE in five out of the eight unbiased samples (Pearson's Correlation, p<0.05), but none of the biased samples. One may therefore consider it to be an additional ‘hidden’ confound, because it is associated with MMSE score in the population of 400 subjects, but it is not significantly associated with MMSE in all the data samples used for model training. However, as we have stated, hidden confounds are unavoidable in practice and they will occur in any experimental evaluation of confounding using real imaging data.

### 5.4 Confounding effects not evaluated in this study

In Section 1 we defined confounds as variables that affect the image data and whose association with the target variable in the training sample differs to that in the population-of-interest. In this paper, we have restricted our attention to cases in which the training sample contains an association between the target variable and the confound, while the population-of-interest does not contain such an association. Our motivation for focusing on this scenario is that in practice, studies often aim to acquire data that is balanced across clinical groups/scores with respect to variables such as age and gender, i.e., such that there is no association between those variables and the target variable, as they are uninterested in such relationships. The evaluation presented in this paper demonstrates what happens if due to e.g., recruitment issues, we are unable to acquire such a data sample, and the potential consequences of training a model with the resulting sample on the predictive accuracy across a population which does not contain such associations. While an exhaustive evaluation of all other possible types of confounding is beyond the scope of this paper, we will now briefly consider other types of confounding that fall within our definition. These include:

1. The training sample does not contain an association between the confound and the target, but the population-of-interest does. An example is if we know that males are more likely to have a lower clinical score than females in our population-of-interest, but the clinical scores are evenly distributed with respect to gender within the training sample.
2. Both the training sample and population-of-interest contain associations between the confound and the target, but in opposing directions. An example is if we know that males are more likely to have a lower clinical score than females in our population-of-interest, but males tend to have higher clinical scores than females within the training sample.
3. Both the training sample and population-of-interest contain associations between the confound and the target in the same direction but to differing degrees. This would occur if e.g., males are only slightly more likely to have a lower clinical score than females in our population-of-interest, but they are much more likely to have a lower clinical score than females within the training sample.

While each of the above situations are slightly different, they all represent a type of confounding (under our definition) due to the differences between the training samples and the population-of-interest. In these cases, different modelling approaches, such as those considered in this paper, may affect predictive performance on unbiased data in potentially different ways to that seen in the current work in each of the different cases. For example we may expect the ‘Images Only’ model to perform better than the model that includes confounds as predictors in case 2), since the explicit inclusion of the confound in the model may learn a relationship between confound and target in the opposite direction to that found in the population-of-interest. Whilst it is also possible that the ‘Images Only’ model would learn this relationship, the degree to which this occurs would likely depend on the extent to which the confound affects the imaging data. On the other hand, in case 3), including the confound as a predictor may be preferable if the ‘Images Only’ model is unable to learn the nature of the relationship between the confound and the target through the effect of the confound on the imaging data.

Dealing with the above types of confounding represents similar challenges to those already discussed in this paper, due to the combination of covariate shift and model misspecification during predictive modelling. One possible approach could be to use an Instance Weighting type of approach in which we include information about the relationship between the confound and the target variable into the weights through Eq. (15) (Section 3.2). However, we leave exploration and analysis of these types of confounding and possible ways of dealing with them, for future research.

### 5.5 Illustrative example

We conclude with an example of how we may deal with a potential confound in practice. Let us consider a case in which we are predicting a measure of clinical depression from imaging data, and we have a drug variable that describes whether a subject is taking a particular drug that affects the brain. With clinical datasets, we often have variables that describe whether a subject is taking medication, and it may be desirable from a neuroscientific perspective to account for these variables in some sense during predictive modelling. If the drug is a successful treatment for depression, we may find a strong negative sample correlation between the depression measure and the drug variable in our dataset that we believe is representative of the population of interest. In this case, we should not consider drug to be a confound, as defined in Section 1, and the sample is unbiased. During predictive modelling we could include the drug variable as an extra input feature, or more conservatively, use the images alone for training the predictive model (Note that we are assuming that there are no other sources of bias in the dataset, e.g., the sample marginal distributions of the depression measure and drug variable are representative of the clinical population-of-interest.). Using a procedure such as image adjustment may potentially worsen the predictive performance on unbiased data, as it would remove the drug effect from the imaging data while preserving its association with the clinical variable ie. it would break the relationship between the imaging data and the clinical variable that we would expect to find in the population. On the other hand, if the drug affects the brain but it isn't a treatment for depression, we would not expect the sample correlation to be present in the population-of-interest. We are then in the presence of confounding, in which the sample is biased by the drug variable. We may then try to use a method such as image adjustment or Instance Weighting to improve predictive performance on unbiased data which does not contain this correlation. Ultimately, deciding whether a variable is a true confound will depend on prior studies of the variable and the exact nature of the population of interest and the data sample, which itself will depend on the recruitment process of the study.

## Figures and Tables

**Fig. A.12:**
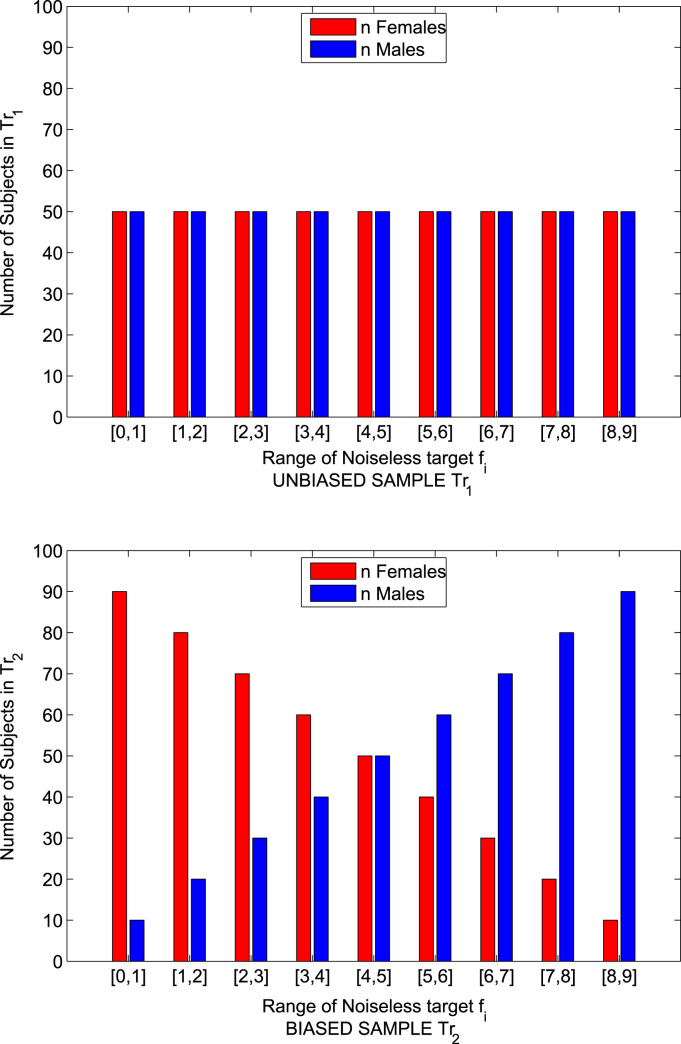
No of subjects by gender over unit intervals of $f_{i}$ in the unbiased training sample ${\mathrm{Tr}}_{1}$ are shown in (a). The corresponding barchart for the biased training sample ${\mathrm{Tr}}_{2}$ is shown in (b).

**Fig. A.13:**
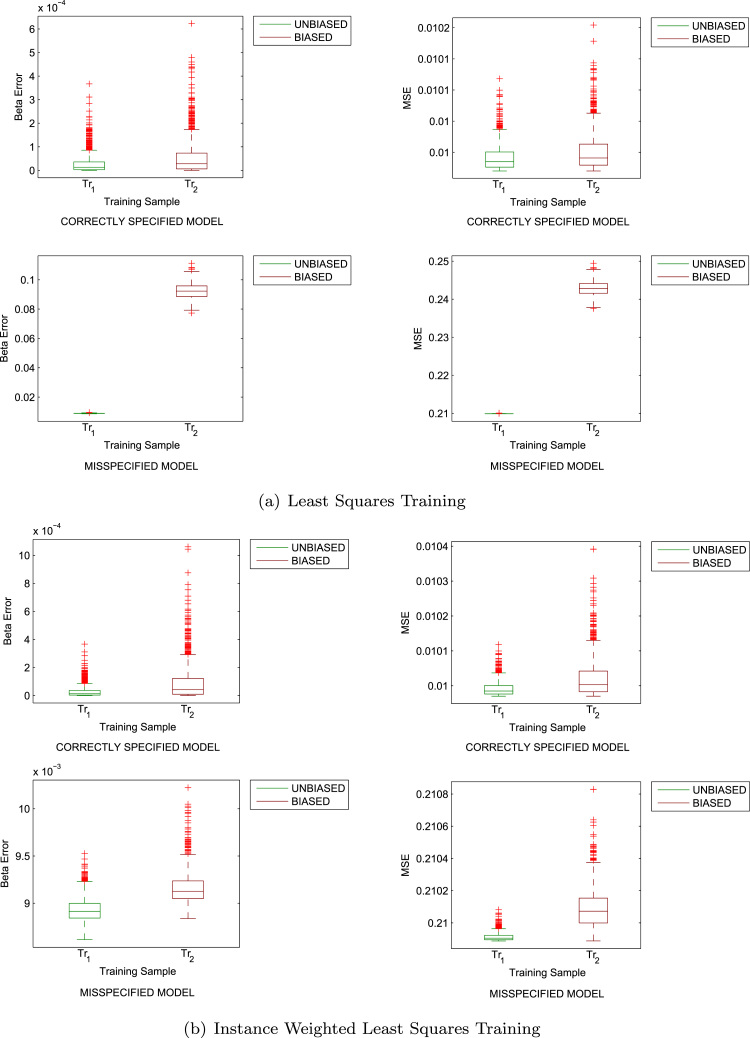
Results using least squares training are shown in (a), while (b) shows results using Instance Weighted least squares. In both figures, the top row shows results when training with a correctly specified model, while the bottom row shows results when training with a misspecified model. The left boxplots in each figure are of ∥β−β^]∥2 while the right boxplots are of the MSE over the test data.

**Fig. A.14:**
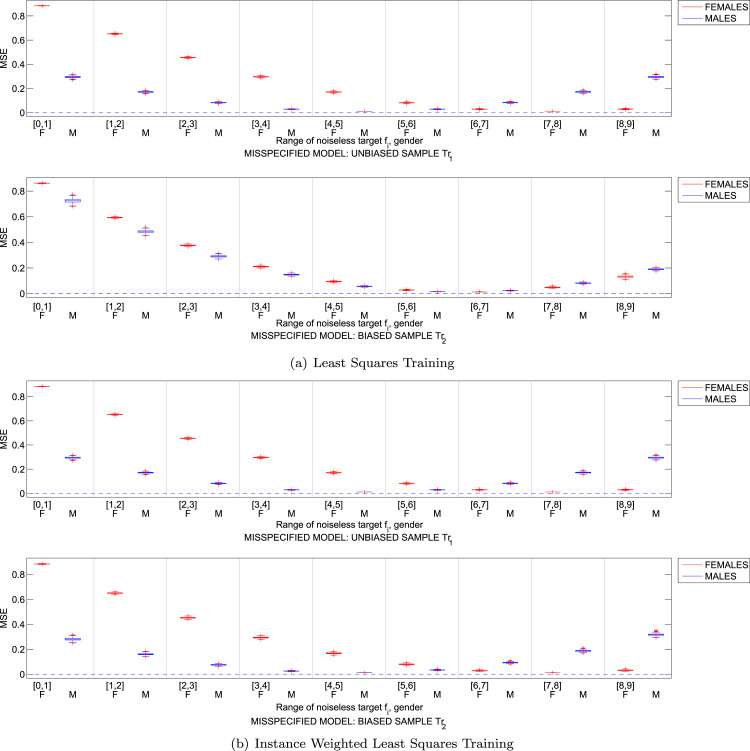
Results using least squares training are shown in (a), while (b) shows results using Instance Weighted least squares. In both figures, the top row shows the MSE as the value of the noiseless target $f_{i}$ changes, for each gender, when training using the unbiased sample ${\mathrm{Tr}}_{1}$ and a misspecified model. The bottom row in each figure shows the corresponding boxplot when using biased sample ${\mathrm{Tr}}_{2}$.

**Fig. A.15:**
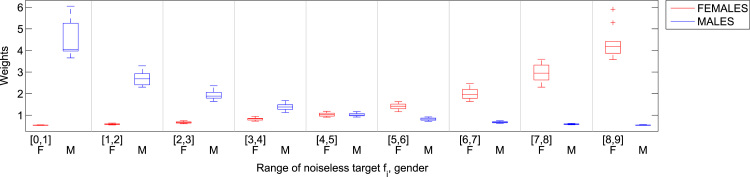
Here we show the weights that are estimated during the Instance Weighting procedure as the value of the noiseless target $f_{i}$ changes for each gender, for one of the biased samples from ${\mathrm{Tr}}_{2}$.

**Table C.7:** Prediction errors for the different models when predicting MMSE.

(a) Biased Training Samples		
Model	*MSE*	*Gb_MSE*
Im. Only	8.03*	8.08*
Adj. Im.	7.90*	8.12*
Im. & C.	8.34*	8.34*
Inst. Wt.	8.26*	8.32*

(b) Unbiased Training Samples		
Model	*MSE*	*Gb_MSE*
Im. Only	7.86*	7.99*
Adj. Im.	7.78*	7.97*
Im. & C.	7.87*	8.01*
Inst. Wt.	7.85*	7.97*

* indicates better performance than chance, p<0.05.

**Table C.8:** Gender-Difference errors for the different models when predicting MMSE.

(a) Biased Training Samples	
Model	*Gd_MSE*
Im. Only	4.95
Adj. Im.	5.45
Im. & C.	5.21
Inst. Wt.	4.83

(b) Unbiased Training Samples	
Model	*Gd_MSE*
Im. Only	4.88
Adj. Im.	4.64
Im. & C.	4.82
Inst. Wt.	4.88

**Fig. C.16:**
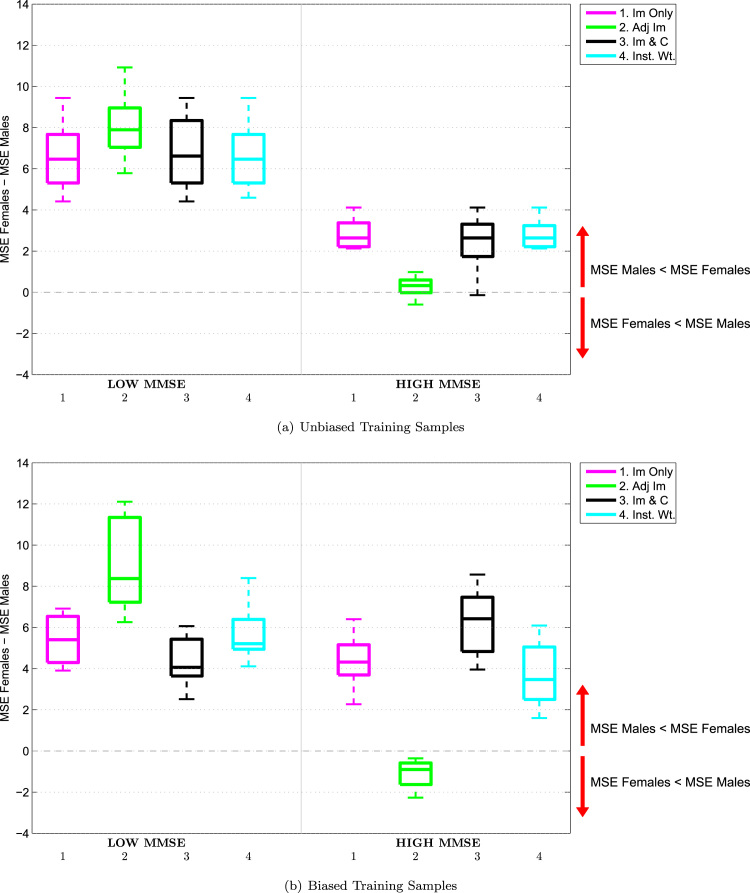
Signed difference between the MSE for females and MSE for males, over the different ranges of the MMSE Score. The data points in each box plot are the 8 predictions performed for a particular model. Results using unbiased training samples are shown in (a), while results using biased training samples are shown in (b). In the biased samples, males tend to have higher MMSE scores.

**Table C.9:** Prediction errors for the different models when predicting age.

(a) Biased Training Samples		
Model	*MSE*	*Sb_MSE*
Im. Only	32.83*	31.94*
Adj. Im.	30.72*	30.16*
Im. & C.	33.63*	32.62*
Inst. Wt.	32.82*	31.98*

(b) Unbiased Training Samples		
Model	*MSE*	*Sb_MSE*
Im. Only	27.65*	26.99*
Adj. Im.	26.88*	26.17*
Im. & C.	27.53*	26.90*
Inst. Wt.	27.65*	26.99*

* indicates better performance than chance, p<0.05.

**Table C.10:** Site-Difference errors for the different models when predicting age.

(a) Biased Training Samples	
Model	*Sd_MSE*
Im. Only	10.29
Adj. Im.	10.03
Im. & C.	13.62
Inst. Wt.	8.10

(b) Unbiased Training Samples	
Model	*Sd_MSE*
Im. Only	8.01
Adj. Im.	6.24
Im. & C.	6.66
Inst. Wt.	8.01

**Fig. C.17:**
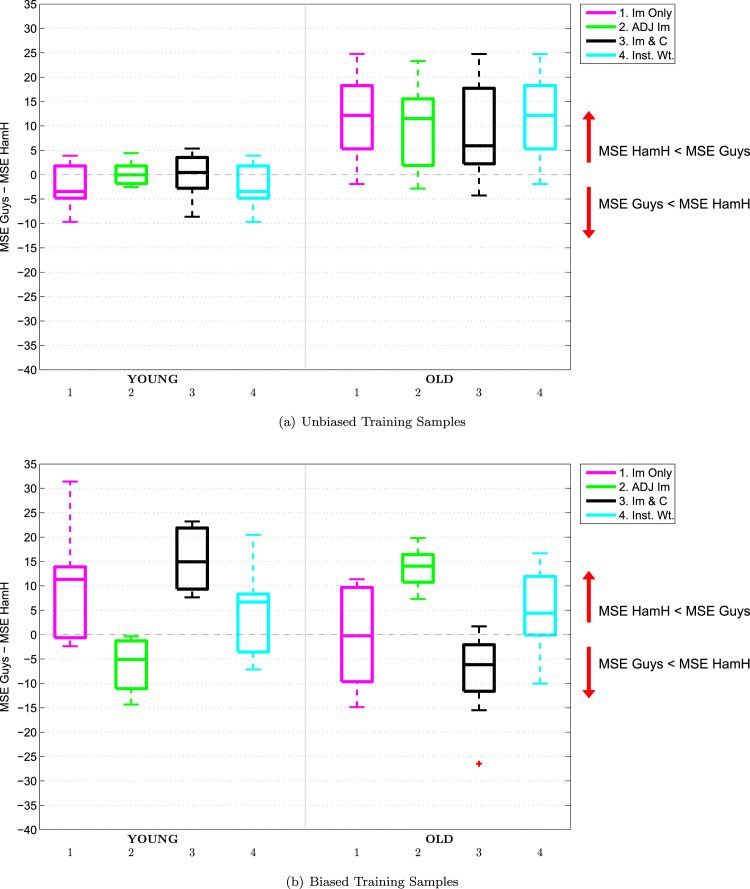
Signed difference between the MSE for Guys and Hammersmith subjects, over the different ranges of age. The data points in each box plot are the 8 predictions performed for a particular model. Results using unbiased training samples are shown in (a), while results using biased training samples are shown in (b). In the biased samples, subjects from Guys tend to be older.

**Table 1:** Partitioning of each fold of test data by range of MMSE score and gender.

	$F_{1}$		$F_{2}$	
n within $R_{l}^{k}$	$R_{1}^{1}$	$R_{2}^{1}$	$R_{1}^{2}$	$R_{2}^{2}$
Females	52	40	49	54
Males	64	44	57	40

**Table 2:** Partitioning of each fold of test data by range of age and site.

	$F_{1}$		$F_{2}$	
n within $R_{l}^{k}$	$R_{1}^{1}$	$R_{2}^{1}$	$R_{1}^{2}$	$R_{2}^{2}$
Guys	48	40	44	50
Hammersmith	26	22	18	24

**Table 3:** Prediction errors for the different models when predicting MMSE.

(a) Biased Training Samples		
Model	MSE	Gb_MSE
Im. Only	8.02*	8.05*
Adj. Im.	7.94*	8.20*
Im. & C.	8.43*	8.42*
Inst. Wt.	8.02*	8.05*

(b) Unbiased Training Samples		
Model	MSE	Gb_MSE
Im. Only	7.58*	7.69*
Adj. Im.	7.64*	7.83*
Im. & C.	7.57*	7.69*
Inst. Wt.	7.61*	7.72*

* indicates better performance than chance, p<0.05.

**Table 4:** Gender-Difference errors for the Prediction of MMSE for the different models.

(a) Biased Training Samples	
Model	Gd_MSE
Im. Only	4.79
Adj. Im.	6.20
Im. & C.	4.84
Inst. Wt.	4.56

(b) Unbiased Training Samples	
Model	Gd_MSE
Im. Only	4.70
Adj. Im.	4.67
Im. & C.	4.71
Inst. Wt.	4.70

**Table 5:** Prediction errors for the different models when predicting age.

(a) Biased Training Samples		
Model	MSE	Sb_MSE
Im. Only	30.08*	29.32*
Adj. Im.	31.66*	31.85*
Im. & C.	31.54*	30.55*
Inst. Wt.	29.91*	29.19*

(b) Unbiased Training Samples		
Model	*MSE*	*Sb_MSE*
Im. Only	25.21*	24.93*
Adj. Im.	25.34*	24.88*
Im. & C.	25.84*	25.56*
Inst. Wt.	25.21*	24.93*

* indicates better performance than chance, p<0.05.

**Table 6:** Site-Difference errors for the prediction of age for the different models.

(a) Biased Training Samples	
Model	*Sd_MSE*
Im. Only	5.47
Adj. Im.	15.97
Im. & C.	8.86
Inst. Wt.	5.28

(b) Unbiased Training Samples	
Model	*Sd_MSE*
Im. Only	7.30
Adj. Im.	6.29
Im. & C.	7.57
Inst. Wt.	7.30

## Appendix A Simulated example

### A.1 Least squares training

We consider a simulated example in which we predict a continuous target y using a single image feature g that is confounded by a binary variable c. To motivate our description in this section we can think of y as a clinical score, g as grey matter volume, and c as gender.

The first step is to generate the three variables for our population-of-interest T, which will be of size 900000. To do this, we firstly define 900000 ‘noiseless’ clinical scores $f_{i}$ by drawing 100000 examples from each of the continuous uniform distributions U(j,j+1) for j=0,…,8. Each U(j,j+1) has the probability density function $q^{U(j,j+1)}$

(A.1) $$q^{U(j,j+1)}(x)=\{\begin{array}{cc} 1 & \mathrm{if}\;x\in [j,j+1]\\ 0 & \mathrm{otherwise}. \end{array}$$

This essentially creates a set of $f_{i}$ that is uniformly distributed over [0,9], while ensuring

(A.2) $$F^{T}\left(l\in [j,j+1]\right)=\frac{1}{9}$$

where $F^{T}$ denotes relative frequency distributions within the population. Eq. (A.2) means that there is an equal number of $f_{i}$ contained within each unit interval [j,j+1]. We set the value of gender c (with c=0 indicating a female) according to

(A.3) $$F^{T}\left(c|f\in [j,j+1]\right)=\frac{1}{2},\;c=0,1$$

so that there is an even split of genders for observations with $f_{i}\in [j,j+1]$, for all values of j. This ensures a minimal correlation between c and f over the population, $\rho =1.1\times 10^{-5}$. The grey matter volume $g_{i}$ is determined as

(A.4) $$g_{i}=f_{i}+3c_{i}$$

giving a positive correlation, ρ=0.50, between g and gender c, i.e., males tend to have a greater grey matter volume than females. Finally, we create the noisy clinical scores $y_{i}$ using

(A.5) $$y_{i}=f_{i}+\epsilon_{i}$$

where each $\epsilon_{i}$ is randomly and independently drawn from N(0,0.1). The correlation between gender c and y over the population is now $\rho =6.5\times 10^{-5}$. The model for the data in the population is therefore

(A.6) $$y_{i}=g_{i}-3c_{i}+\epsilon_{i}$$

and the binary variable c, gender, will be the confounding variable in our experiments: It is associated with grey matter volume g, but it is not associated with the clinical score y in our population-of-interest.

We take half of the observations in the resulting data to create an unbiased test sample according to

(A.7) $$F^{\mathrm{Test}}\left(f\in [j,j+1]\right)=\frac{1}{9}\;F^{\mathrm{Test}}(c|f\in [j,j+1])=\frac{1}{2}$$

as in the population, while the remaining observations will be sampled for training. This ensures that the relative frequency distributions $F^{\mathrm{Test}}(c)$, $F^{\mathrm{Test}}(f)$ and $F^{\mathrm{Test}}(c|f)$ are approximately equal to those of T, i.e., the test sample is representative of the population and is therefore unbiased. We then perform two experiments in which g and c are used as input features to predict the clinical scores y of the unbiased test sample.

In the first experiment, we test whether bias in the training sample will affect the learning of the predictive model when the model is correctly specified. Firsly, we create an unbiased training sample ${\mathrm{Tr}}_{1}$ consisting of 900 observations sampled ‘randomly’ from the non-test data according to

(A.8) $$F^{{\mathrm{Tr}}_{1}}\left(f\in [j,j+1]\right)=\frac{1}{9}\;F^{{\mathrm{Tr}}_{1}}(c|f\in [j,j+1])=\frac{1}{2}$$

so that the training sample is representative of the population, with minimal correlation between c and y (Fig. A.12(a)).

We then fit the linear model

(A.9) $$y=\beta_{1}g+\beta_{2}c$$

to give estimates for $\beta =[\beta_{1},\beta_{2}]$ using linear least squares, i.e., we find the parameters [β^1,β^2] that minimise the least-squares loss function

(A.10) [β^1,β^2]=argmin∑i=1n1n(yi−(β1gi+β2ci))2

This has the closed-form solution

(A.11) [β^1,β^2]T=(XTX)−1XTy.

Here, the i th row of the design matrix X consists of $\left[g_{i},c_{i}\right]$. Our predictive function is then

(A.12) f(g,c)=β^1g+β^2c

which is therefore based on the approach in Eq. (7), in which the image features and confounds are used as predictors. The function f is then used to predict the clinical scores of the unbiased test sample. We then produce a biased training sample ${\mathrm{Tr}}_{2}$ by sampling 900 observations from the non-test data according to

(A.13) $$F^{{\mathrm{Tr}}_{2}}\left(f\in [j,j+1]\right)=\frac{1}{9}\;F^{{\mathrm{Tr}}_{2}}(c|f\in [j,j+1])=\left\{ \begin{array}{cc} 1-\frac{j+1}{10} & ,c=0\\ \frac{j+1}{10} & ,c=1. \end{array}\right.$$

Although the relative frequency distributions $F^{{\mathrm{Tr}}_{2}}(c)$, $F^{{\mathrm{Tr}}_{2}}(f)$ are still approximately equal to that of the population, the distribution $F^{{\mathrm{Tr}}_{2}}(c|f)$ differs because there is no longer an even split of genders for observations with $f_{i}\in [j,j+1]$ (Fig. A.12(b)). This results in a strong positive marginal correlation, ρ≈0.51, between c (gender) and y in the training sample ${\mathrm{Tr}}_{2}$, in contrast to the population T for which there is a minimal correlation between these variables. The training sample ${\mathrm{Tr}}_{2}$ is therefore a biased sample in which c is a true confound. We now fit the linear least squares model of Eq. (A.9) with this biased training sample, and use the estimate β^=[β1^,β2^] to predict the unbiased test sample with Eq. (A.12) once more.

The top left of Fig. 13(a) shows the $L_{2}$ norm of the difference between the estimated model coefficients and the true ones, ∥β−β^∥2, over 1000 repetitions of the unbiased/biased sample training. We can see that β has been well estimated using both the unbiased training sample ${\mathrm{Tr}}_{1}$ and the biased sample ${\mathrm{Tr}}_{2}$, resulting in accurate predictions of the test sample in each case, as shown in the top right of Fig. 13(a). Clearly the ‘spurious’ marginal correlation between c and y in ${\mathrm{Tr}}_{2}$ has not affected model estimation: The predictions have *not* been ‘driven’ by the marginal association of the confound and the target in the biased sample ${\mathrm{Tr}}_{2}$, which would suggest that confounding has not affected the predictive modelling in this case. We should note, however, that our model estimation procedure i.e the linear least squares fit of Eq. (A.16), is the optimal one given that the data was produced using Eq. (A.6), which is a linear model with homoskedastic guassian noise. In practice we will not know a-priori how our data was generated so will not be able to specify the correct predictive model as we have done here.

Our second experiment now investigates how bias in the training sample will affect predictive modelling when the predictive model is misspecified. To do this, we add an intercept term of 1 to the target variable so that the data is generated according to the model

(A.14) $$y_{i}=g_{i}-3c_{i}+1+\epsilon_{i}.$$

We use the same unbiased and biased samples of training data, ${\mathrm{Tr}}_{1}$ and ${\mathrm{Tr}}_{2}$, as in the first experiment, and use linear least squares to fit the data. However, for both ${\mathrm{Tr}}_{1}$ and ${\mathrm{Tr}}_{2}$ we perform the fit using the model in Eq. (A.9) so that the intercept term in Eq. (A.14) is not modelled. We are therefore attempting to fit a wrong, or misspecified, model to the data. The estimates β1^,β2^ are then used to predict the test data using Eq. (A.12) once more.

The bottom row of Fig. 13(a) shows the errors in the estimated model coefficients and the prediction errors over 1000 repetitions of the unbiased/biased sample training, with the misspecified model. If we compare these results with those in the first experiment, shown in the top row of Fig. A.13, we can see a worsening in estimated model coefficients and predictive accuracy for both ${\mathrm{Tr}}_{1}$ and ${\mathrm{Tr}}_{2}$. This is to be expected, as using a poorer model is bound to worsen predictive performance in this example. In contrast to the previous experiment, however, the models produced using the biased sample ${\mathrm{Tr}}_{2}$ are now consistently worse than those using the unbiased sample ${\mathrm{Tr}}_{1}$: The model misspecification has had an additional adverse effect on predictive modelling with the biased training sample. This differs from the first experiment, where the unbiased and biased training samples performed similarly with considerable overlap in their error distributions over the 1000 repetitions.

In order to further investigate the differences between training with a biased and unbiased sample under model misspecification, Fig. 14(a) shows the mean-squared errors for males and females as the value of the noiseless target $f_{i}$ changes, when using ${\mathrm{Tr}}_{1}$ (top row of Fig. 14(a)) and ${\mathrm{Tr}}_{2}$ (bottom row of Fig. A.14) for training. We can see that with the unbiased sample ${\mathrm{Tr}}_{1}$, males are predicted more accurately than females for lower values of the clinical score, while with higher values, females are predicted more accurately than males. This is a consequence of model misspecification, and in neuroimaging where the correct model is not known, such effects are unavoidable. If we now consider the corresponding boxplot for ${\mathrm{Tr}}_{2}$, we can see that, while the MSE for females is quite similar to that for ${\mathrm{Tr}}_{1}$, males tend to be predicted far worse than with ${\mathrm{Tr}}_{1}$ for lower values of the clinical score and slightly better for high values. This corresponds with the distribution of gender/clinical score in ${\mathrm{Tr}}_{2}$, in which males tend to have higher values of the clinical score. The combination of misspecification and bias in the training sample has therefore caused the resulting model to focus on the examples that occur more frequently in the training sample. This results in the poor model estimates obtained when using ${\mathrm{Tr}}_{2}$ and the corresponding poor predictions on the unbiased test sample which is representative of the population-of-interest and therefore does not contain any association between gender and clinical score. The practical implications of this simulation are that if we wish to train models for prediction from neuroimaging data, bias in the training sample may adversely affect prediction accuracy on test samples taken from the population-of-interest, i.e., the population over which we wish to apply the predictive model. This will be a consequence of model misspecification which in practice is unavoidable.

### A.2 Instance weighted training

Here we perform experiments with the simulated data described in A.2, using Instance Weighting. As described in 3.2, 3.3, this requires that we solve a weighted version of the problem. Here, this means that instead of solving the least-squares problem of Eq. (A.10), we solve the weighted version

(A.15) [β^1,β^2]=argmin∑i=1n1nwi(yi−(β1gi+β2ci))2

where the weights wi=P^S(yi)P^S(yi|ci) need to be estimated from the data in the training sample. We obtain the weights using Gaussian Process Regression in the same way as described in Section 4.2.4, i.e., we fit two independent gaussian processes to give P^S(y) and P^S(y|c) and then evaluate these densities at each point i in the sample to determine the weights wi=P^S(yi)P^S(yi|ci). Please see that section for further details. The solution to Eq. (A.15) is then given by

(A.16) [β^1,β^2]T=(XTWX)−1XTWy

where W is a diagonal matrix with entries $w_{i}$. The predictive function is then as in Eq. (A.12), i.e.,

(A.17) f(g,c)=β^1g+β^2c.

As in our earlier experiments, we will perform the estimation using both unbiased samples ${\mathrm{Tr}}_{1}$, and biased samples ${\mathrm{Tr}}_{2}$, in two scenarios. Firstly, we will apply this procedure to the case where the predictive model is correctly specified, i.e., when the data is produced according to

(A.18) $$y_{i}=g_{i}-3c_{i}+\epsilon_{i}.$$

Secondly, we will perform estimation under model misspecification, i.e., when the data is produced according to

(A.19) $$y_{i}=g_{i}-3c_{i}+1+\epsilon_{i}$$

which contains an intercept term that is not modelled. We will once more be interested in how model misspecification changes the relative performance of the predictive models when training with biased samples as opposed to unbiased samples.

The top left of Fig. 13(b) shows the $L_{2}$ norm of the difference between the estimated model coefficients and the true ones, ∥β−β^∥2, over 1000 repetitions of the unbiased/biased sample training, using the weighted fitting with the correctly specified model. We can see that β has been well estimated using both the unbiased training sample ${\mathrm{Tr}}_{1}$ and the biased sample ${\mathrm{Tr}}_{2}$, resulting in accurate predictions of the test sample in each case, as shown in the top right of Fig. 13(b). This is therefore similar to the results using (unweighted) least squares fitting shown in the top row of Fig. 13(a), where including the confound as a predictor did not reduce predictive performance when the model is correctly specified. The bottom row of Fig. 13(b) shows how the weighted model performs under model misspecification. If we compare this to the bottom row of Fig. 13(a) we can see that, with unbiased training samples ${\mathrm{Tr}}_{1}$, model misspecification has caused the unweighted and weighted fits to give similar reductions in predictive performance and parameter estimates. However, if we now look at the results using biased training samples ${\mathrm{Tr}}_{2}$ and weighted estimation shown in the bottom row of Fig. 13(b), we can see that the predictive performance and parameter estimates are quite similar to those with ${\mathrm{Tr}}_{1}$, with a degree of overlap in the error distributions over the 1000 repetitions. This shows that by using the instance weighting, we can obtain a model that is similar in predictive accuracy to that which would have been obtained if we had trained the model using an unbiased sample. This contrasts with the results shown in the bottom row of Fig. 13(a) using the unweighted estimation, where bias in the sample causes the predictive model to degrade considerably. Fig. 14(b) shows the mean-square errors for males and females as the value of the noiseless target $f_{i}$ changes, when using ${\mathrm{Tr}}_{1}$ (top) and ${\mathrm{Tr}}_{2}$ (bottom) for training, with the weighted estimation and a misspecified model. We can see that the distribution of errors for males and females is quite similar whether we use biased or unbiased samples, which again contrasts with the corresponding results using the unweighted approach, shown in Fig. 14(a), where bias in the sample causes a large change in the distribution of errors by gender over the target range.

Finally, in order to give an intuition of what is happening during the weighting, Fig. A.15 shows a boxplot of the determined weights as the value of the noiseless target $f_{i}$ changes for each gender, when using one of the biased samples from ${\mathrm{Tr}}_{2}$ for training. We can see that the weights for females increase as the target increases, while for males the weights decrease with an increasing value of the target. If we refer to Fig. A.12(b), which shows the distribution of gender by $f_{i}$ in samples from ${\mathrm{Tr}}_{2}$, we can see that the weighting is placing less emphasis on subjects that are over-represented (with respect to the population-of-interest) in the sample, and greater emphasis on subjects that are under-represented. This results in the improved performance of the Instance Weighted least squares training compared to standard least squares training when we have a biased training sample and the model is misspecified.

## Appendix B Optimization of marginal likelihood for heterogenous gaussian likelihood

Here we describe how we implemented the optimization of the Marginal Likelihood of the heteregenous Gaussian Likelihood:

(B.1) $$P(y_{i}|f_{i})=\frac{1}{\sigma_{i}\sqrt{2\pi }}e^{-\frac{{\left(y_{i}-f_{i}\right)}^{2}}{2\sigma_{i}^{2}}}$$

where $\sigma_{i}=\frac{\sigma }{\sqrt{w_{i}}}$, and $w_{i}$ are the instance weights. The marginal likelihood for a Gaussian Process with kernel K and the above likelihood function is then

(B.2) $$\log Z=-\frac{1}{2}y^{T}{\left(K(\theta )+\sigma^{2}W\right)}^{-1}y\;-\frac{1}{2}\log\left|K(\theta )+\sigma^{2}W\right|-\frac{n}{2}\log2\pi$$

where W is a diagonal matrix with entries $w_{i}$. This can be obtained directly by observing that $y∼N(K,\sigma^{2}W)$. Although the above is the exact expression for the Marginal Likelihood, we optimize it using Laplacian inference for ease of implementation within the GPML toolbox. This should be exactly equivalent to optimizing the exact expression given above. To do this, we require the following derivatives which are used during the optimization routine:

(B.3) $$\frac{\partial }{\partial f_{i}}\log(P(y_{i}|f_{i}))=\frac{y_{i}-f_{i}}{\sigma_{i}^{2}}\;\frac{\partial^{2}}{\partial f_{i}^{2}}\log(P(y_{i}|f_{i}))=-\frac{1}{\sigma_{i}^{2}}\;\frac{\partial^{3}}{\partial f_{i}^{3}}\log(P(y_{i}|f_{i}))=0\;\frac{\partial }{\partial \log\sigma }\log(P(y_{i}|f_{i}))=\frac{{\left(y_{i}-f_{i}\right)}^{2}}{\sigma_{i}^{2}}-1\;\frac{\partial }{\partial \log\sigma }\frac{\partial }{\partial f_{i}}\log(P(y_{i}|f_{i}))=2\frac{\left(f_{i}-y_{i}\right)}{\sigma_{i}^{2}}\;\frac{\partial }{\partial \log\sigma }\frac{\partial^{2}}{\partial f_{i}^{2}}\log(P(y_{i}|f_{i}))=\frac{2}{\sigma_{i}^{2}}.$$

## Appendix C Results with low dimensional data

In this section we present results where the input features are low dimensional region-of-interest (ROI)-based features rather than voxel-based features. We create these features by averaging the voxel-based features over the ROIs contained in the AAL atlas (Tzourio-Mazoyer et al., 2002), after reslicing the atlas to the same dimensions as the aligned image data. The resulting input features are of dimension 116. The same models and evaluation scheme as described in 4.2, 4.3 were used.

### C.0.1 Results with ADNI data

Table C.7 shows the error measures using biased and unbiased training samples. As with the high-dimensional features, all models perform better than chance according to MSE and Gb_MSE, and predictive performance is reduced when there is bias in the training samples. Table C.7(a) shows that the ‘Images & Confounds’ model is the worst performing model with biased training samples, although the ‘Instance Weighted’ model also appears to perform poorly in comparison with the ‘Adjusted Images’ and ‘Images Only’ models. A possible reason for this could be due to an increase in variance of this model, as weighting effectively reduces the size of the training sample (Shimodaira, 2000). Table C.8 shows the gender difference errors for each model, and we can see that with biased samples, the ‘Adjusted Images’ and ‘Images & Confounds’ models seem to give bigger differences than the other models. If we now look at the boxplot in Fig. C.16, we can see that bias in the training samples tends to change the distribution of the prediction errors in a similar fashion to that found with the high dimensional data. For example, if we focus on the ‘Images Only’ model in Fig. 16(b), we can see that for subjects with low MMSE scores, the signed difference between the MSE for females and that for males decreases compared to the corresponding measure in Fig. 16(a). Conversely, for subjects with high MMSE scores, the signed difference between the MSE for females and that for males increases compared to the corresponding measure in Fig. 16(a). These shifts are amplified with the ‘Images & Confounds’ model, although the ‘Instance Weighted’ model appears to have slighly smaller shifts than the ‘Images Only’ model. It is possible that the weighting of examples has caused the ‘Instance Weighted’ model to give a distribution of prediction errors that is more similar to that when using unbiased samples with respect to the gender differences, although the overall accuracies are worse. Lastly, we find that the ‘Adjusted images’ model seems to shift the gender differences in the opposite direction to the other models, overcompensating for bias in the sample as with the voxel-based features.

### C.0.2 Results with IXI data

Table C.9 shows the error measures using biased and unbiased training samples. All models perform better than chance according to MSE and Sb_MSE, and predictive performance is again reduced when there is bias in the training samples. Table C.9(a) shows that the ‘Images & Confounds’ model is the worst performing model with biased training samples. In contrast to the results with the high dimensional features, however, the ‘Adjusted Images’ model performs better than the other models. This may be due to the particular model used during the adjustment procedure being more appropriate at the ROI level, perhaps because of a reduction in noise due to the averaging of features within each ROI. The ‘Instance Weighted’ model performs similarly to the ‘Images Only’ model in terms of predictive performance. If we now look at the site-difference errors shown in Table C.10, we can see that the ‘Instance Weighted’ model gives the smallest value for this measure with the biased training samples, while the ‘Adjusted Images’ and ‘Images & Confounds’ models give the smallest site-difference errors with unbiased training samples. If we now consider the corresponding boxplot in Fig. C.17, we can see that bias in the training samples tends to once more change the distribution of the prediction errors in a similar fashion to that found with the high dimensional data. For example, if we focus on the ‘Images Only’ model in Fig. 17(b), we can see that for young subjects the signed difference between the MSE for Guys and Hammersmith subjects increases compared to the corresponding result in Fig. 17(a). Conversely, for older subjects this signed difference decreases compared to the corresponding result in Fig. 17(a). As before, these shifts are amplified with the ‘Images & Confounds’ model. Interestingly, the shifts for the ‘Instance Weighted’ model are not as pronounced as for the ‘Images Only’ model, indicating that while the prediction accuracies for both models are quite similar, the distribution of predictive accuracies with respect to the site differences, is not as affected by bias in the training samples for the ‘Instance Weighted’ model as for the ‘Images Only’ model.

## References

[bib1] Abdulkadir, A., Ronneberger, O., Tabrizi, S.J., Kloppel, S., 2014. Reduction of confounding effects with voxel-wise Gaussian process regression in structural MRI, Proceedings - 2014 International Workshop on Pattern Recognition in Neuroimaging, PRNI 2014, pp. 1–4. http://dx.doi.org/10.1109/PRNI.2014.6858505.

[bib2] Austin P.C., Stuart E.A. (2015). Moving towards best practice when using inverse probability of treatment weighting (IPTW) using the propensity score to estimate causal treatment effects in observational studies. Stat. Med..

[bib3] Bickel S., Brückner M., Scheffer T. (2009). Discriminative learning under covariate shift. J. Mach. Learn. Res..

[bib4] Brown M.R.G., Sidhu G.S., Greiner R., Asgarian N., Bastani M., Silverstone P.H., Greenshaw A.J., Dursun S.M. (2012). ADHD-200 global competition: diagnosing ADHD using personal characteristic data can outperform resting state fMRI measurements. Front. Syst. Neurosci..

[bib5] Chu C., Ni Y., Tan G., Saunders C.J., Ashburner J. (2011). Kernel regression for fMRI pattern prediction. NeuroImage.

[bib6] Cole S.R., Hernán M.A. (2008). Constructing inverse probability weights for marginal structural models. Am. J. Epidemiol..

[bib7] Doyle O.M., Ashburner J., Zelaya F.O., Williams S.C.R., Mehta M.a., Marquand a.F. (2013). Multivariate decoding of brain images using ordinal regression. NeuroImage.

[bib8] Dukart J., Schroeter M.L., Mueller K. (2011). Age correction in dementia-matching to a healthy brain. PloS One.

[bib9] Duvenaud, D., 2014. Automatic Model Construction with Gaussian Processes. (Ph.D. thesis), Computational and Biological Learning Laboratory, University of Cambridge.

[bib10] Focke N.K., Helms G., Kaspar S., Diederich C., Tόth V., Dechent P., Mohr A., Paulus W. (2011). Multi-site voxel-based morphometry - Not quite there yet. NeuroImage.

[bib11] Good P. (2005). Permutation, Parametric, and Bootstrap Tests of Hypotheses.

[bib12] Gretton A., Smola A.J., Huang J., Schmittfull M., Borgwardt K., Schölkopf B. (2009). Covariate shift by kernel mean matching. Dataset Shift Mach. Learn..

[bib13] Hirano K., Imbens G.W.G., Berkeley U.C. (2004). The propensity score with continuous treatments. Appl. Bayesian Model. Causal Inference Incomplete-Data Perspect..

[bib14] Imai K., Ratkovic M. (2014). Covariate balancing propensity score. J. R. Stat. Soc.: Ser. B (Stat. Methodol.).

[bib15] Kostro D., Abdulkadir A., Durr A., Roos R., Leavitt B.R., Johnson H., Cash D., Tabrizi S.J., Scahill R.I., Ronneberger O., Klöppel S. (2014). Correction of inter-scanner and within-subject variance in structural MRI based automated diagnosing. NeuroImage.

[bib16] Linn, K.A., Gaonkar, B., Doshi, J., Davatzikos, C., Shinohara, R.T., 2015. Addressing Confounding in Predictive Models with an Application to Neuroimaging, The International Journal of Biostatistics, ISSN 1557–4679, http://dx.doi.org/10.1515/ijb-2015-0030, URL 〈http://www.degruyter.com/view/j/ijb.ahead-of-print/ijb-2015-0030/ijb-2015-0030.xml?Format=INT〉. 10.1515/ijb-2015-0030 PMC5154735 26641972

[bib17] Marquand A., Howard M., Brammer M., Chu C., Coen S., Miranda J.Mourão. (2010). Quantitative prediction of subjective pain intensity from whole-brain fMRI data using Gaussian processes. NeuroImage.

[bib18] Pan S.J., Tsang I.W., Kwok J.T., Yang Q. (2011). Domain adaptation via transfer component analysis. IEEE Trans. Neural Netw..

[bib19] Rao, A., Monteiro, J.M., Ashburner, J., Portugal, L., Fernandes, O., Oliveira, L.D., Pereira, M., Mourao-Miranda, J., 2015. A Comparison of Strategies for Incorporating Nuisance Variables into Predictive Neuroimaging Models. 2015 International Workshop on Pattern Recognition in NeuroImaging, pp. 61–64. http://dx.doi.org/10.1109/PRNI.2015.28, URL 〈http://ieeexplore.ieee.org/lpdocs/epic03/wrapper.htm?Arnumber=7270848〉.

[bib20] Rasmussen C.E. (2006). Gaussian Processes for Machine Learning.

[bib21] Scholkopf B., Smola A.J. (2002). Learning with Kernels: Support Vector Machines, Regularization, Optimization, and Beyond.

[bib22] Shimodaira H. (2000). Improving predictive inference under covariate shift by weighting the log-likelihood function. J. Stat. Plan. Inference.

[bib23] Stonnington C.M., Chu C., Klöppel S., Jack C.R., Ashburner J., Frackowiak R.S.J. (2010). Predicting clinical scores from magnetic resonance scans in Alzheimer's disease. NeuroImage.

[bib24] Sugiyama M., Suzuki T., Nakajima S., Kashima H., Von Bünau P., Kawanabe M. (2008). Direct importance estimation for covariate shift adaptation. Ann. Inst. Stat. Math..

[bib25] Sugiyama M., Krauledat M., Müller K.F. (2007). Covariate shift adaptation by importance weighted cross validation. J. Mach. Learn. Res..

[bib26] Takao H., Hayashi N., Ohtomo K. (2013). Effects of the use of multiple scanners and of scanner upgrade in longitudinal voxel-based morphometry studies. J. Magn. Reson. Imaging.

[bib27] Tzourio-Mazoyer N., Landeau B., Papathanassiou D., Crivello F., Etard O., Delcroix N., Mazoyer B., Joliot M. (2002). Automated anatomical labeling of activations in SPM using a macroscopic anatomical parcellation of the MNI MRI single-subject brain. NeuroImage.

[bib28] Vapnik V. (1992). Principles of risk minimization for learning theory. Adv. Neural Inf. Process. Syst..

[bib29] Wachinger, C., Reuter, M., Domain Adaptation for Alzheimer’s Disease Diagnostics, Neuroimage, ISSN 10538119, http://dx.doi.org/10.1016/j.neuroimage.2016.05.053. 10.1016/j.neuroimage.2016.05.053 PMC4983466 27262241

[bib30] Young J., Modat M., Cardoso M.J., Mendelson A., Cash D., Ourselin S. (2013). Accurate multimodal probabilistic prediction of conversion to Alzheimer's disease in patients with mild cognitive impairment. NeuroImage: Clin..

